# Recovery-Primed Stimulation: A Proposed Framework for Controlled Triggering of Healing-Related Physiology in Low-Grade Chronic Inflammation

**DOI:** 10.7759/cureus.114334

**Published:** 2026-08-11

**Authors:** Wilfried Van Moorleghem, Mark Mikhail, Peter Besselink, Luis Chin, Alejandra Velazquez, Melia Matuz Velasquez, Diego A Montelongo Mercado

**Affiliations:** 1 Research and Development, Macmed, Puerto Escondido, MEX; 2 Research, Imperial College Healthcare NHS Trust, London, GBR; 3 Engineering, Memory Metal Holland (MMH), Twente, NLD; 4 Regenerative Medicine, Universidad Popular Autónoma del Estado de Puebla, Puebla, MEX

**Keywords:** chronic inflammation, low-grade chronic inflammation, recovery primed medicine, recovery primed stimulation, recovery primed triggering, systemic inflammation, triggering physiology

## Abstract

Low-grade chronic inflammation (LGCI) is considered a heterogeneous, persistent, high-burden biological state rather than a single disease entity. In LGCI, inflammation-related pathways can reinforce each other, creating a self-sustaining inflammatory state. This creates a treatment-design problem: stimulation delivered into LGCI biology that is already primed by persistent inflammatory drivers and has not fully returned to a resolved resting state may produce the expected, wanted biological effects, such as braking, resolution, repair, or regulatory recovery, but may also worsen LGCI by adding inflammatory drive to already active inflammatory pathway loops. This unwanted effect may include nuclear factor kappa B (NF-κB) or inflammasome reactivation, cytokine-loop amplification, flare response, increased autonomic-inflammatory pressure, temporal summation, or adverse carryover.

Recovery-primed stimulation (RP) is a proposed framework for controlled triggering of healing-related physiology followed by recovery-compatible repetition. In RP, a biochemical, biophysical, or combined input is used as a controlled trigger of wanted healing-related physiology, and repetition is delayed until the induced wanted response has had time to unfold and unwanted carryover is absent or acceptable. The hypothesis is that RP may better fit LGCI physiology than dense repetition and may improve the balance between wanted and unwanted effects. RP extends this controlled-triggering and recovery-compatible-repetition logic across biochemical, biophysical, or combined inputs and across multiple LGCI-relevant target pathway axes. It is presented as an interdisciplinary technology platform for controlled biological triggering in low-threshold, slow-recovery biology.

## Introduction and background

Certain cofactors, many related to modern life, can promote low-grade chronic inflammation (LGCI) and contribute to major chronic disease fields that represent leading causes of disability and mortality worldwide [1-4]. Because these cofactors differ between individuals, LGCI is heterogeneous and may present through different pathway-dominant phenotypes.

Inflammaging, the age-associated form of LGCI, is linked to aging-related conditions and multimorbidity and has been described as a broad feature of modern human aging [2,3,5,6]. These links do not make LGCI a single treatable disease, but they do show why persistent low-grade inflammatory biology is an important research target.

Despite major progress in anti-inflammatory pharmacology, there is still no widely accepted general treatment strategy that reliably resolves heterogeneous LGCI across phenotypes [7]. Conventional approaches can be clinically valuable, but they often suppress or block selected inflammatory outputs [8,9].

LGCI may persist because several interacting pathway systems continue to reinforce each other, including nuclear factor kappa B (NF-κB) signaling, NOD-, LRR-, and pyrin domain-containing protein 3 (NLRP3) inflammasome priming and activation, interleukin (IL)-23/IL-17 amplification, gut-axis signaling, autonomic-inflammatory loops, oxidative and mitochondrial stress, tissue damage, and impaired resolution [10-13].

This creates a treatment-design problem. In low-threshold LGCI biology, a biological input that is useful under recovered conditions may produce unstable or unwanted effects if it is delivered into a pathway system that is already primed, incompletely recovered, or close to an activation threshold. Repeated stimulation may then fail to improve the wanted effect or may add inflammatory carryover through NF-κB reactivation, inflammasome priming, cytokine-loop amplification, autonomic-inflammatory pressure, flare response, or cumulative burden [14-18].

The biological problem is therefore not only how to reduce inflammatory output but also how to engage endogenous braking, resolution, repair, barrier recovery, autonomic recovery, metabolic recovery, and regulatory reset mechanisms without interrupting them too early. In acute inflammation, these processes unfold over time and support a return toward homeostasis. In LGCI, they may be delayed, incomplete, or repeatedly disrupted by persistent cofactors, tissue damage, inflammatory memory, or premature restimulation. This is especially relevant to endogenous NF-κB feedback systems mediated by inhibitor of NF-κB (IκB) proteins and tumor necrosis factor alpha-induced protein 3 (A20) and to specialized pro-resolving mediator biology, which supports active resolution rather than passive decay [10,12,19,20].

Recovery-primed stimulation (RP) is a proposed framework for controlled triggering of healing-related physiology followed by recovery-compatible repetition. In RP, a biochemical, biophysical, or combined input is used as a controlled trigger to induce a wanted endogenous response, such as inflammatory braking, resolution signaling, repair signaling, barrier recovery, autonomic recovery, metabolic recovery, or another phenotype-relevant regulatory response [12,21].

In this framework, timing is essential but is not the biological objective. Its role is to place the next trigger after the induced response has had enough time to unfold, useful responsiveness has returned, and unwanted carryover is absent or acceptable.

This article is a hypothesis-driven conceptual narrative review. A narrative design was selected to synthesize heterogeneous mechanistic and translational evidence across biological pathways and stimulation modalities into a testable conceptual framework. Literature was selected for mechanistic relevance to RP in LGCI, with emphasis on peer-reviewed studies and reviews addressing chronic low-grade inflammation, inflammaging, NF-κB signaling, inflammasome biology, inflammatory resolution, gut-barrier biology, autonomic-inflammatory regulation, allostatic load, recovery physiology, and stimulation-based intervention frameworks. The selected literature was used to build a pathway-based rationale and to identify testable design variables for future protocol-specific validation.

This paper presents RP as a framework hypothesis for adapting repeated biological triggering to LGCI physiology. The aim is not continuous suppression of inflammatory output, but controlled engagement of endogenous braking, resolution, repair, and return toward homeostasis. The framework predicts that recovery-compatible repetition may improve the balance between wanted and unwanted effects compared with dense, arbitrary, or premature repetition, but this prediction requires pathway-specific, modality-specific, protocol-specific, and population-specific validation.

## Review

Methodology: literature identification and conceptual synthesis

This article is a hypothesis-driven conceptual narrative review, not a systematic or scoping review. The literature was collected iteratively between January 2015 and March 2026, primarily through Google Scholar searches. Relevant articles were examined in full or in pertinent part, and their reference lists were used to identify additional mechanistically informative sources. Potentially relevant publications were retained in a working Zotero library containing more than 15,000 references accumulated during the broader research program. This number represents the broader working library, not a defined set of studies formally screened for or included in this review.

Searches and source selection evolved as biological relationships and pathway interactions were examined. The review therefore did not use a preregistered protocol, a single fixed exhaustive search string, duplicate independent screening, a formal disagreement-resolution procedure, or a standardized study-level risk-of-bias instrument. Sources cited in the article were selected through an author-directed, relevance-based process according to their ability to inform LGCI biology, inflammatory braking and resolution, recovery-control mechanisms, pathway-response dynamics, or a defined component of the RP hypothesis. Evidence was weighted qualitatively according to mechanistic relevance, model type, proximity to human LGCI, and whether it supported established biology, mechanistic inference, or author-proposed RP hypotheses.

Because direct studies of the integrated RP framework are not yet available, the evidence was organized by biological pathway and synthesized qualitatively through hypothesis-generating mechanistic bridging. Findings from relevant human, animal, and cellular studies, including studies primarily addressing other diseases, tissues, models, or interventions, were used only where they supported a specific mechanistic or temporal statement relevant to the framework. Such evidence was treated as indirect or contextual and was not pooled, treated as equivalent, or presented as direct evidence of RP safety or therapeutic effectiveness. Differences in species, model, phenotype, disease context, tissue, modality, dose, endpoint, and observation time were considered limitations to transferability.

No meta-analysis, meta-regression, statistical pooling, or quantitative heterogeneity assessment was performed. Experimental observation times were interpreted descriptively within their respective biological and experimental contexts and were not treated as functional recovery periods or quantitatively comparable measures. Consequently, neither the RP framework nor its proposed repetition intervals was quantitatively derived from the reviewed literature. Any specific intervals proposed for future investigation are author-generated research hypotheses, not pooled estimates, established recovery periods, or evidence-based clinical recommendations.

The cited literature supports the individual biological mechanisms to varying degrees, whereas their integration into RP and any resulting safety or therapeutic effectiveness remain hypotheses requiring direct experimental and clinical validation. Direct studies reporting positive, neutral, contradictory, or harmful outcomes of the complete RP framework are not currently available.

Recovery primed stimulation and the RP technology platform

Basic RP Definition

RP is a proposed physiology-guided framework for using repeated biological triggers to engage wanted endogenous responses, including inflammatory braking, resolution signaling, repair signaling, regulatory recovery, barrier recovery, autonomic recovery, metabolic recovery, or another phenotype-relevant recovery-control response. Actual responses may be wanted, neutral, unwanted, or harmful and therefore require monitoring and adaptation.

The RP recovery interval is the biological response period after a trigger, during which the wanted biology is allowed to unfold. It is selected in relation to the wanted-effect window and recovery state of the targeted biology so that repetition occurs only when useful responsiveness has returned and unwanted carryover is absent or acceptable.

A controlled trigger does not necessarily produce the intended response. Observed effects may be wanted, neutral, unwanted, or unexpected, including potentially harmful effects. RP therefore requires predefined wanted and unwanted effects, monitoring of the observed response and recovery state, and protocol adaptation, pausing, or stopping when the wanted response is absent or unacceptable effects occur.

RP Variables

Pulse drive refers to pulse characteristics, including pulse amplitude, pulse width, and other modality-specific pulse variables. Stimulation refers to the applied pulse together with its recovery interval because the biological effect of the pulse depends on the pathway state into which the next pulse is delivered.

Recovery interval refers to the time between two pulses. In RP, this interval is selected in relation to the wanted-effect window and, when relevant, the recovery or reset time of the targeted biology from an activated, refractory, adapted, or incompletely recovered state. During this interval, wanted mechanisms may unfold toward their useful or maximal effect window. The next stimulation is then applied to re-engage the wanted response while limiting unwanted carryover.

A wanted effect is a predefined favorable effect of the stimulation, such as inflammatory braking, resolution signaling, repair signaling, barrier recovery, autonomic recovery, metabolic recovery, or another phenotype-relevant recovery-control response. An unwanted effect is a predefined, unfavorable effect of the stimulation, such as non-targeted pathway activation, temporal summation, adverse carryover, flare response, or cumulative burden.

The RP effect means the biological effects observed after RP-timed stimulation. It includes observed wanted and unwanted effects. Under the RP hypothesis, well-selected recovery intervals are expected to improve the RP effect by increasing wanted effects, reducing unwanted effects, or improving the balance between the two.

In RP, a recovery-compatible interval does not mean that all biological activity has returned to baseline. It means that the previous wanted response has unfolded sufficiently, unwanted carryover is absent or acceptable, and the targeted pathway system is able to respond usefully to the next pulse. Recovery compatibility is therefore judged by the predefined wanted effect, unwanted effect, phenotype, safety signals, and recovery markers, not by clock time alone.

Central RP Principle: Triggering Healing Physiology and Allowing Recovery to Unfold

The central RP principle is that a controlled trigger should be followed by enough recovery time for the induced wanted biology to unfold. These wanted responses may be direct or downstream effects and may include endogenous inhibitory, braking, resolution, repair, or regulatory mechanisms. The next stimulation is then timed to the recovery state of the targeted pathway system, not to an arbitrary frequency or fixed schedule.

The aim is to increase the cumulative wanted effects obtained per pulse. This means that the recovery interval should be long enough for the wanted effects to unfold, but not so long that these effects become too small or disappear before the next pulse.

The RP recovery interval is therefore not inactive rest time. It is a crucial, active part of the stimulation sequence. The proposed hierarchy is as follows: healing-related physiology first; controlled triggering second; induced biological reaction third; recovery fourth; and timing fifth. Timing is the protocol tool that protects and repeats the biological reaction. The recovery interval matters because it gives the induced braking, resolution, repair, and reset processes time to perform the intended work before the next trigger.

Multimodal Scope

RP is multimodal because biochemical, biophysical, or combined inputs can produce a physiological response [22,23]. Biochemical inputs may include pharmacological [24], nutritional, metabolic, or other molecule-based inputs.

Biophysical inputs may include mechanical, electromagnetic, or other physical forms of stimulation [24-26]. The modality determines how the pathway system is engaged.

The modalities are not considered therapeutically equivalent, and evidence from one modality is not treated as validation of another. Biological evidence is organized by pathway, while modalities represent different potential means of engaging the targeted pathway system. The recovery-compatible interval determines whether repeated triggering remains aligned with the wanted-effect window and recovery state of the targeted biology.

Multiaxis Scope

RP is multiaxis because different modalities can influence multiple physiological pathway axes when triggering and recovery timing are matched to the wanted-effect window of the targeted pathway system. RP inputs can influence more than one axis, but the affected axes depend on the input used and the biological state in which it is applied [27-31].

In LGCI, this multiaxis scope is important because inflammatory persistence may involve interactions with nuclear factor kappa B (NF-κB), the inflammasome, interleukin (IL)-23/IL-17, the gut-axis, autonomic, oxidative, mitochondrial, tissue-damage, and resolution-control pathways. RP is therefore not limited to one pathway target, but the selected target axes should be defined before testing. Inflammation does not resolve by suppression alone; it requires endogenous braking, resolution, cleanup, repair, and return toward homeostasis. RP therefore starts from physiology: select a controlled trigger, induce a wanted biological reaction, allow the recovery response to unfold, and repeat only when the targeted pathway system is again recovery-compatible.

RP Technology Platform

The core of the RP Technology Platform is controlled triggering of healing-related physiology followed by recovery-compatible repetition. Timing remains an essential protocol variable, but it is not the biological objective. Its role is to align the next trigger with the recovery state and wanted-effect window of the targeted pathway system.

The RP Technology Platform is modality-adaptable because the same recovery-compatible design principle can be applied to different biological inputs, provided that the selected input produces a measurable physiological response. It is also condition-adaptable because the same architecture can be adjusted to different pathway systems, phenotypes, safety limits, and intended wanted effects.

**Figure 1:**
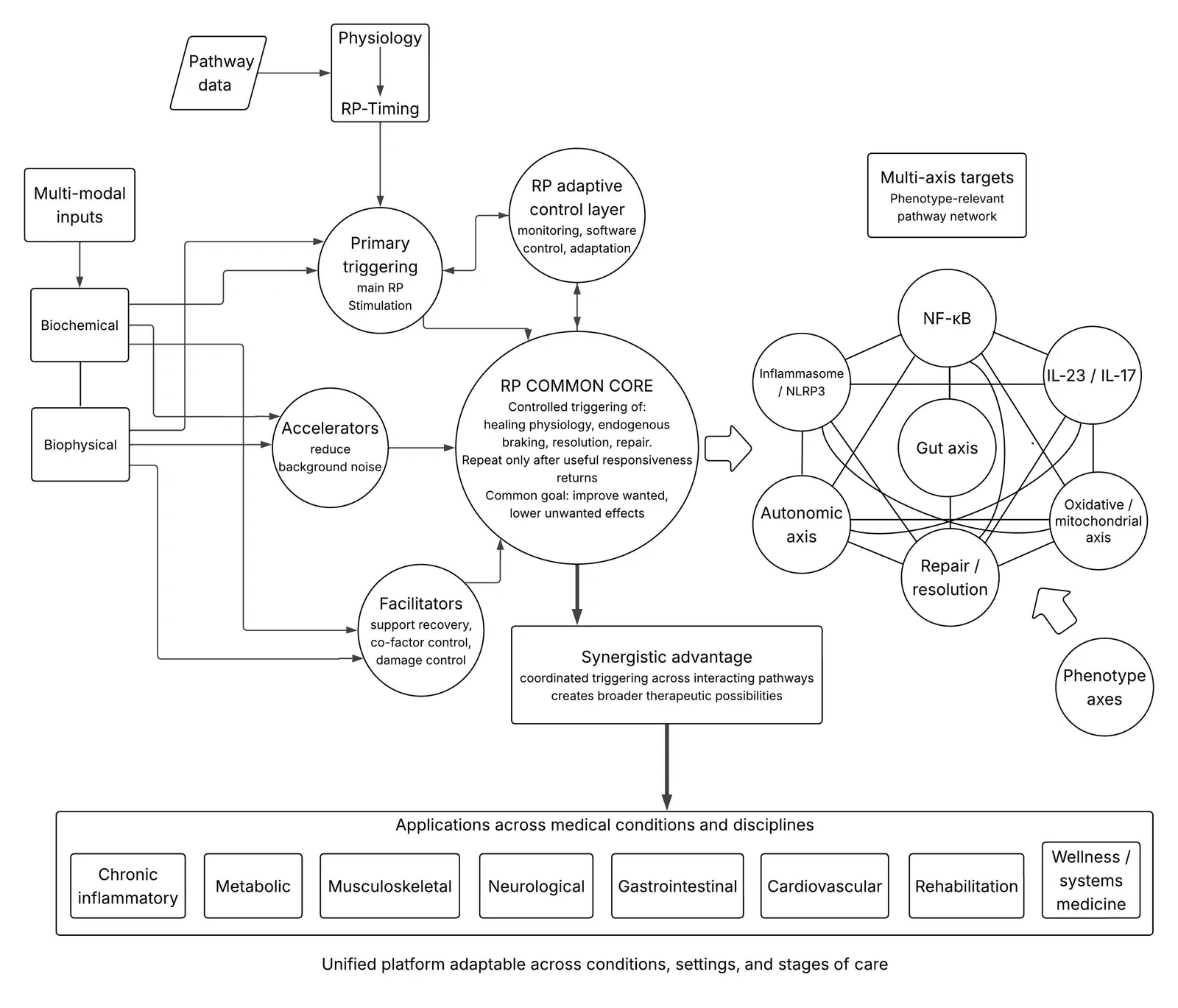
RP Technology Platform. This author-developed conceptual RP Technology Platform schematic presents a framework for coordinating controlled primary triggering, accelerators, facilitators, monitoring, and recovery-compatible repetition across multimodal inputs and LGCI-relevant pathway systems. The cited sources support the biological and stimulation elements represented in the schematic, including biophysical and biochemical stimulation, dose- and recovery-dependent biological responses, allostatic-load biology, autonomic regulation, NF-κB feedback control, NOD-, LRR-, and pyrin domain-containing protein 3 (NLRP3) inflammasome biology, oxidative and endoplasmic reticulum (ER) stress signaling, and inflammation-related biochemical modulation [10-17,21,32-35]. RP: recovery primed stimulation, LGCI: low-grade chronic inflammation, ER: endoplasmic reticulum.

This study applies the RP Technology Platform to LGCI (Figure 1). The principle may not be limited to LGCI; however, extension to neurological, cardiovascular, musculoskeletal, metabolic, rehabilitation, gastrointestinal, wellness, and systems-medicine applications remains hypothetical and requires separate indication- and modality-specific evidence [32-35]. In each case, repeated biological triggering would aim to improve wanted effects while limiting unwanted effects by matching repetition to recovery biology.

Low-grade chronic inflammation

Why LGCI Matters

Certain social, environmental, and lifestyle cofactors can promote LGCI. LGCI-related mechanisms are implicated in major chronic disease fields, including cardiovascular disease, stroke, cancer, diabetes, chronic kidney disease, nonalcoholic fatty liver disease, autoimmune disease, arthritis, and neurodegenerative disorders. Collectively, these have been recognized as the most significant cause of death in the world today, causing 50% of all worldwide mortality [3]. LGCI in aging, often discussed as inflammaging [2,4], is linked to aging-related conditions; reduced lifespan; lower physiological resilience; impaired recovery, equilibrium, and mobility; higher vulnerability to stressors; and loss of reserve [2,3,5,6].

Examples of disease areas in which LGCI-related mechanisms have been discussed in the literature are presented in the Appendices. The economic values in the table represent total disease-specific U.S. burden estimates and are not intended as LGCI-attributable cost estimates but as an illustration of the scale of selected chronic condition fields in which inflammatory biology is implicated [36-52].

LGCI State

LGCI is used here as an operational physiological concept, not as a single disease entity. In this article, LGCI refers to persistent, low-level inflammation-related pathway activity that may arise gradually from chronic or recurrent biological drivers or may persist after an incompletely resolved inflammatory response. It can involve cytokine signaling, inflammasome activation, tissue-amplification loops, gut-barrier dysfunction, metabolic stress, autonomic dysregulation, impaired resolution, environmental or nutritional burden, or combinations of these mechanisms [1,3,53].

For RP protocol design, LGCI presentations may be provisionally categorized by evidence of primed inflammatory signaling, low response threshold, slow recovery after biological challenge, or stimulation-sensitive adverse carryover. These are proposed RP-relevant response categories, not established clinical LGCI phenotypes. Priming refers to pathway systems that are already transcriptionally, inflammasome-related, metabolically, autonomically, or immunoregulatory activated or near activation [1,3,11,54,55].

Low-threshold biology refers to a state in which smaller inputs may produce larger or less stable responses because inflammatory, stress-related, or allostatic systems are already burdened. Biologically, this means that baseline pathway activity is elevated, inhibitory reserve is reduced, sensitization or priming is present, and the system is closer to an activation threshold than in a recovered resting state [15,35,56,57]. Slow-recovery biology refers to the delayed return of braking, resolution, repair, immune-regulatory, barrier, autonomic, or metabolic systems after stimulation [58-62]. Stimulation-sensitive biology refers to a phenotype in which repeated biological input may produce adverse carryover, flare response, unstable response, or reduced RP effects if delivered before sufficient recovery has occurred. Examples include obesity-associated inflammation [63] or metabolic inflammation [46], inflammaging-related loss of reserve [64], gut-axis-associated inflammatory burden [65], post-infectious or post-stress inflammatory states [66], and selected stable inflammatory disorders in which recovery timing is being investigated as an adjunctive protocol variable.

In an LGCI state, repeated stimulation may interact with incompletely recovered systems and produce lower RP effects than the same input under more recovered conditions [1,12,15,56,59,60]. The following section describes the main LGCI-relevant inflammatory pathway axes: NF-κB, NLRP3, the IL-23/IL-17 axis, and the gut-axis loop.

These axes can drive or maintain persistent inflammatory activity through self-reinforcing inflammatory loops. The timing relevance of each pathway for RP-based LGCI management or durable correction is then described.

Canonical NF-κB Pathway in LGCI: p50/p65 Dimer

The canonical NF-κB pathway is included because it is a central inflammatory signaling hub through which multiple LGCI-relevant inputs can converge. The p50/p65 heterodimer is the major canonical NF-κB dimer relevant to LGCI. Both subunits contribute to DNA binding, while the p65/RelA subunit provides major transcriptional activation capacity [67].

**Figure 2:**
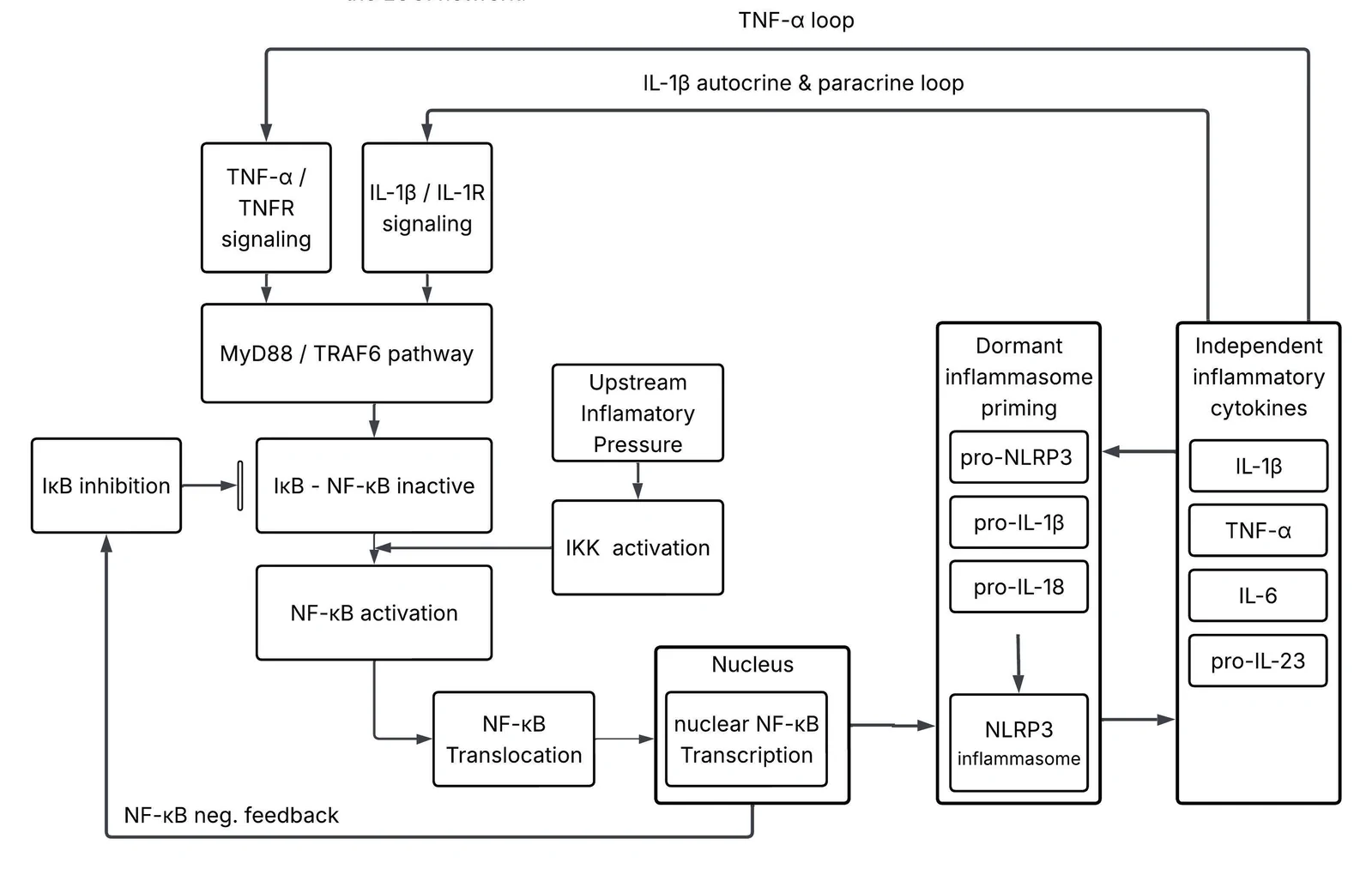
Conceptual NF-κB pathway-loop schematic. This author-created conceptual schematic depicts established NF-κB pathway-loop biology relevant to LGCI. The cited sources support the canonical p50/p65 NF-κB pathway, IκB-controlled cytoplasmic restraint, IκB kinase (IKK)-dependent activation, NF-κB nuclear translocation and transcription, NF-κB-dependent NLRP3 and pro-IL-1β priming, tumor necrosis factor alpha (TNF-α)/interleukin-1 beta (IL-1β) autocrine and paracrine reinforcement, and IκBα/IκBε/A20 feedback control [59,67-73].

Through canonical NF-κB signaling, cytokines, microbial products, damage-associated molecular patterns (DAMPs) sensed by pattern-recognition receptors, cellular stress, tissue damage, and metabolic stress can reinforce inflammatory transcription and cytokine-loop activity. This provides one route by which gut-axis signals, microbial products, DAMPs, and tissue damage connect to NF-κB-driven inflammatory transcription and by which NF-κB can prime inflammasome readiness. NF-κB-activating inputs increase NLRP3 and pro-IL-1β expression and place the cell in a stronger inflammasome-ready state. NF-κB priming is an activation-enabling step that can link low-grade inflammatory signaling to stronger inflammasome output (Figure 2) [54,68]. 

Resting cells: In resting cells, p50/p65 is maintained mainly in an inactive IκB-bound cytoplasmic pool. This resting state is dynamic rather than static. Homeostatic IκB metabolism controls basal NF-κB activity, and basal IKK activity can phosphorylate NF-κB-bound IκBα, targeting it for ubiquitin-dependent degradation. Resting NF-κB control is therefore best described as a calm, dynamic, inhibitor-controlled steady state with very low basal NF-κB activity rather than as a fully inactive, locked state [70,71].

Acute inflammatory activation, NF-κB oscillation, and shutdown: During acute inflammatory activation, strong upstream signaling activates the IKK complex. IKK phosphorylates a larger fraction of NF-κB-bound IκBα, leading to ubiquitination and proteasomal degradation of the inhibitor. This releases larger amounts of p50/p65, allowing nuclear translocation, binding to NF-κB response elements in DNA, and stronger transcription of inflammatory and feedback-control genes, thereby initiating a strong inflammatory response. Newly synthesized IκBα contributes to early termination by entering the nucleus, binding NF-κB, displacing it from DNA, and promoting nuclear export. While upstream inflammatory drive and IKK activity remain present, repeated IκBα degradation and resynthesis support NF-κB nuclear-cytoplasmic oscillations.

As the acute inflammatory drive decreases and IKK activity falls, delayed IκBε feedback helps dampen NF-κB oscillations, stabilize signaling dynamics, and limit inflammatory gene expression. Together, IκBα and IκBε negative feedback can terminate the acute NF-κB transcriptional phase once the acute upstream drive has resolved and IKK activity falls back toward basal levels [59,73-75].

Persistent LGCI, no shutdown: In established LGCI, persistent low-grade inflammatory signaling may arise from self-sustaining cytokine loops, tissue damage, DAMPs, microbial-product exposure, metabolic stress, mitochondrial stress, or other LGCI-maintaining drivers. These inputs may keep the canonical NF-κB pathway slightly biased toward activation rather than a full return to a resting, inhibited state.

Low-level upstream signaling can repeatedly activate IKK at low or moderate levels, increase IκB turnover, reduce effective IκB-bound restraint of p50/p65, and increase the probability of low-grade nuclear NF-κB activity. The result is not a high-peak acute oscillatory response but a persistent, low-level smoldering NF-κB state: not full acute inflammation, but not normal resting homeostasis either [59,76].

NF-κB endogenous brakes relevant for RP: In Alves et al., IκBε remained strongly detectable at 64 hours in one experimental model [58,59]. This model-specific detectability does not establish continued functional braking, a general recovery window, or an RP repetition interval. Other NF-κB-relevant brakes are also important for RP timing. Sirtuin 1 (SIRT1) can inhibit NF-κB-related inflammatory signaling through transcriptional regulation and p65 deacetylation-related mechanisms, with experimental readouts extending beyond 72 hours [77-80]. NF-κB/IκB feedback timing is supported by experimental mechanistic studies, but the longer IκBε observation at 64 hours is model- and stimulus-specific. Its relevance to RP is indirect: it supports allowing post-trigger feedback to unfold but does not establish a general 64-hour recovery interval, an LGCI protocol, or RP effectiveness.

Cyclic adenosine monophosphate (cAMP)-protein kinase A (PKA) signaling is an intracellular signaling/brake pathway. It can inhibit NF-κB-related inflammation and can support slower macrophage resolution functions, including reprogramming, efferocytosis, and IL-10 production, with experimental readouts extending beyond 48 hours [81].

NLRP3 Pathway

**Figure 3:**
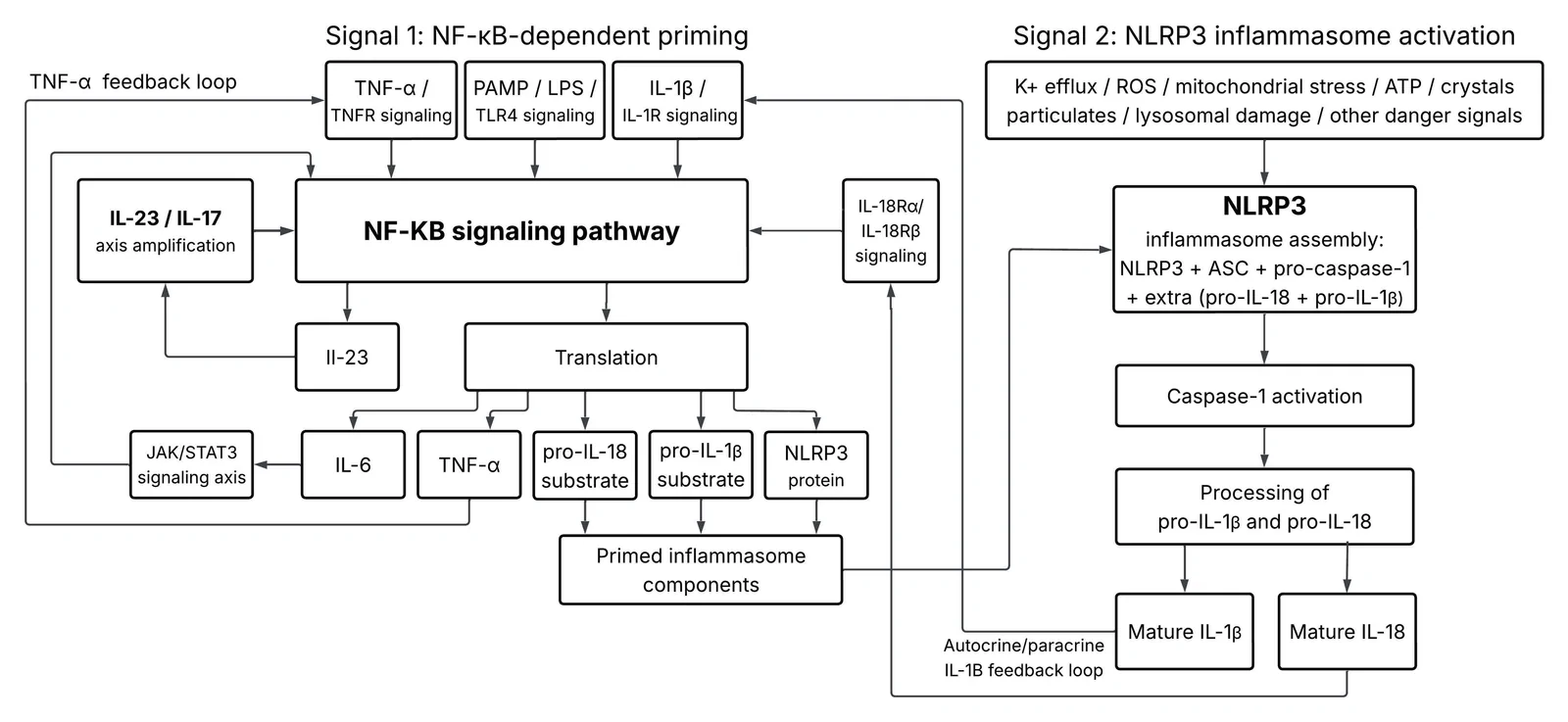
Conceptual NLRP3 inflammasome pathway schematic (with NF-κB priming). Author-created conceptual schematic summarizing established NLRP3 inflammasome biology, including NF-κB-dependent priming, danger-signal activation, IL-1β/IL-18 maturation, and inflammatory feedback into NF-κB signaling [11,13,54,68,82].

NLRP3 can maintain persistent LGCI through a priming and activation loop. NF-κB signaling primes NLRP3 by increasing NLRP3 and pro-IL-1β. A second signal, such as adenosine triphosphate (ATP), ionic flux, mitochondrial dysfunction, reactive oxygen species (ROS), lysosomal damage, crystals, particulates, or DAMPs, can then activate inflammasome assembly. Activated NLRP3 promotes caspase-1 activation, maturation of IL-1β and IL-18, and IL-1β feedback into NF-κB signaling. This links NF-κB and NLRP3 into a self-reinforcing inflammatory loop (Figure 3) [11,13,82].

NLRP3 also has brakes and recovery-control processes that can require long windows. cAMP-PKA signaling is an intracellular brake pathway for NLRP3-related inflammation. PKA can directly inhibit NLRP3 activation by phosphorylating NLRP3 and reducing its ATP-dependent activation step. It can also support slower macrophage resolution functions, including reprogramming, efferocytosis, and IL-10 production, with experimental readouts extending beyond 48 hours [81].

Efferocytosis and DAMP clearance are downstream resolution and cleanup functions, mainly executed by macrophages and other phagocytes. By removing apoptotic cells and reducing DAMP release, they lower reactivation pressure on NF-κB and NLRP3 and support IL-10/transforming growth factor beta (TGF-β)-related resolution signaling. Pro-resolving acute-phase windows have been reported up to 7 days [83,84].

c-Jun N-terminal kinase (JNK)-linked control of NLRP3 is complex because JNK1 has been described both in NLRP3 priming/activation and in later proteasomal removal of NLRP3-related components. For RP timing, the relevant point is the delayed disposal process: E3-ligase-mediated ubiquitination and proteasomal degradation can reduce the pool of inflammasome-ready NLRP3, limit renewed inflammasome assembly, lower IL-1β and IL-18 maturation potential, and help return the pathway toward a less activation-prone state. This is better interpreted as a delayed NLRP3 recovery-control process, not as an immediate anti-inflammatory effect, with a reported recovery-control window of around 36 hours [85-87].

IL-23/IL-17 Axis

**Figure 4:**
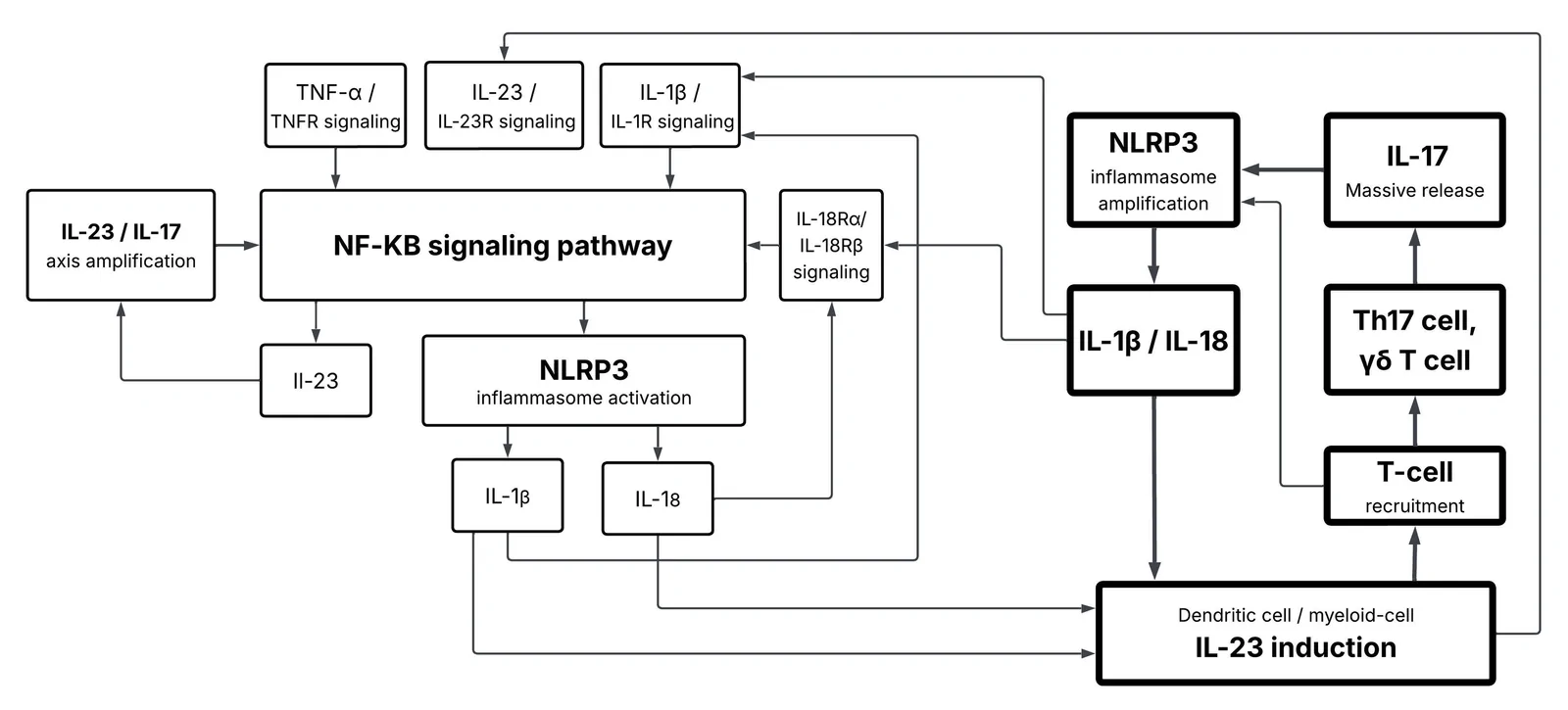
Conceptual IL-23/IL-17 axis pathway-loop schematic. An author-created conceptual schematic summarizing established IL-23/IL-17 axis inflammatory amplification biology, and its interaction with NF-κB, NLRP3, IL-1β/IL-18 signaling, and tissue-damage-associated inflammatory feed-forward loops [11,13,54,68,82,88-90].

The IL-23/IL-17 axis can contribute to persistence of LGCI-related inflammatory activity through tissue-amplification loops (Figure 4). NF-κB-linked cytokine signaling, including IL-1β, IL-18, and TNF-α, can support IL-23 production. IL-23 supports IL-17-producing immune responses, and IL-17 can increase neutrophil recruitment, tissue inflammation, barrier injury, and matrix damage [88]. Tissue damage can release DAMPs that feed back into NF-κB and NLRP3 activation, linking innate priming to tissue-level inflammatory persistence [89,90]. 

IL-23/IL-17 axis brakes relevant for RP: The IL-23/IL-17 axis is limited by upstream neuroimmune cytokine-damping recovery-control processes. Cholinergic anti-inflammatory signaling can reduce upstream TNF-α and IL-1β inputs that support IL-23-driven IL-17 amplification, with experimental anti-inflammatory effects reported up to 48 hours [91].

T helper 17 (Th17)-regulatory T-cell (Treg)-forkhead box P3 (FoxP3) immune-regulatory rebalancing shifts the IL-23/IL-17 axis away from Th17/IL-17 tissue-amplifying output and toward FoxP3-associated regulatory T-cell containment. This process is slower because it involves transcriptional and phenotypic T-cell reprogramming as part of adaptive immune recovery. IL-17 and regulatory-output changes are commonly assessed over approximately 72 hours to 5 days [92,93].

Gut Axis Loop

The gut-barrier/microbiome-immune containment axis can contribute to the persistence of LGCI-related inflammatory activity through a dysbiosis-barrier-dysfunction loop. Dysbiosis can reduce short-chain fatty acid support, weaken immune tolerance, and increase intestinal permeability.

**Figure 5:**
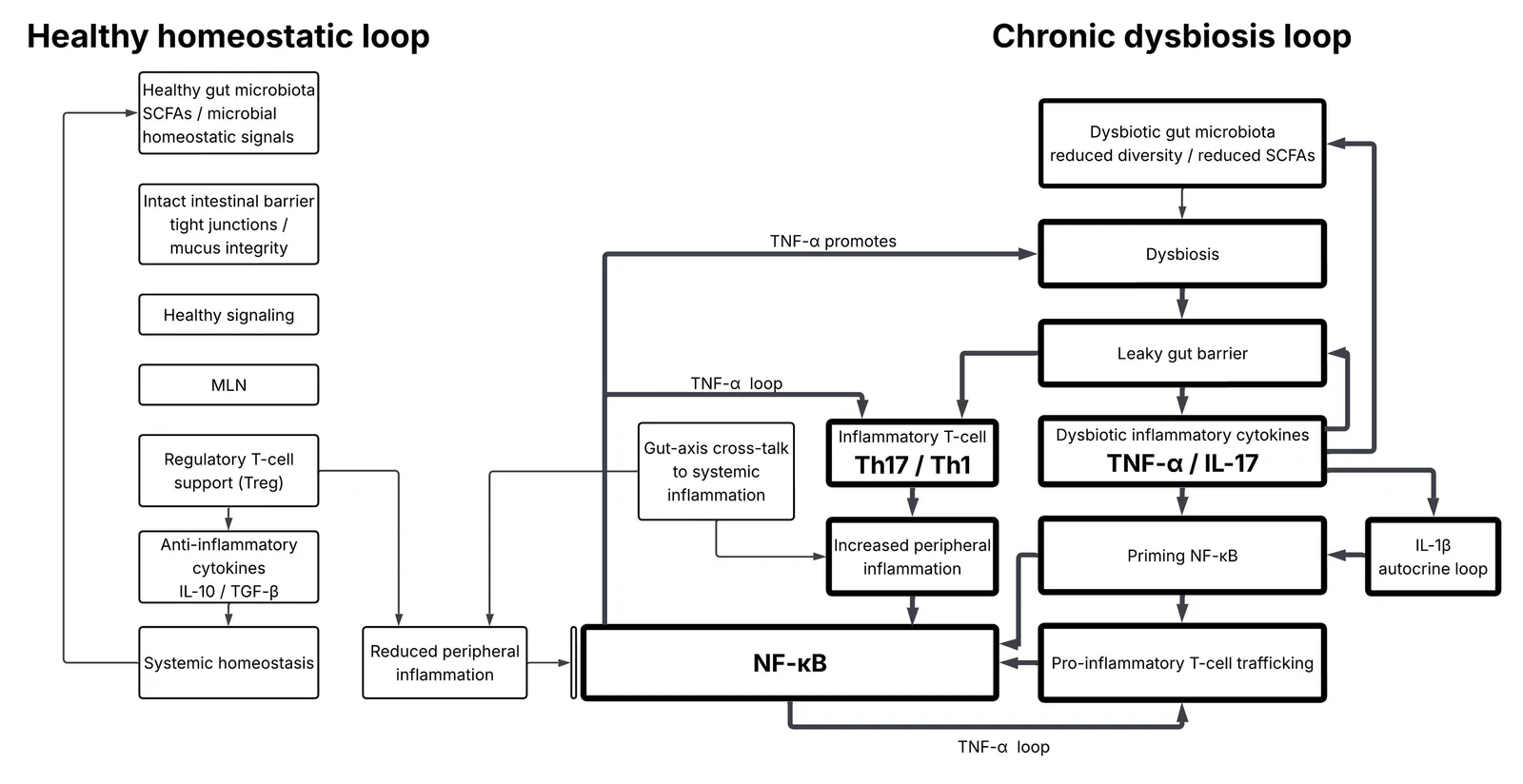
Conceptual gut-axis pathway-loop schematic showing healthy gut homeostasis and dysbiosis-driven gut-to-systemic inflammatory amplification. This author-created conceptual schematic depicts established gut-axis inflammatory-loop biology. The cited sources support the role of gut microbiota, short-chain fatty acids, intestinal barrier integrity, tight-junction and mucus function, microbial-product translocation, metabolic endotoxemia, TLR4/NF-κB priming, dysbiosis-associated cytokine signaling, Th17/Th1 inflammatory responses, Treg/IL-10/TGF-β regulatory support, and gut-to-systemic inflammatory cross-talk relevant to LGCI [65,84,94-99]. LGCI: low-grade chronic inflammation.

Microbial products such as lipopolysaccharide (LPS) can then cross the barrier, activate Toll-like receptor 4 (TLR4), prime NF-κB and NLRP3, and increase TNF-α, IL-1β, and IL-6 signaling. These cytokines can further damage barrier integrity, allowing continued microbial-product exposure and persistent inflammatory drive (Figure 5) [65,94-98].

Gut-axis recovery includes restoration of epithelial barrier integrity, immune tolerance, microbial containment, and microbiota-related regulatory signaling. Relevant mechanisms include butyrate-Treg-IL-10 signaling, IL-22-mediated epithelial repair, AhR signaling, bile acid receptor signaling, secretory IgA, and recovery of microbiota composition. Cholinergic anti-inflammatory signaling and early cytokine suppression may operate across 24- to 48-hour windows [91].

Barrier repair and immune reprogramming, including butyrate-Treg-IL-10, IL-22, AhR, and bile-acid receptor signaling, commonly require multiday windows. Microbial containment and dysbiosis-related recovery, including changes in secretory IgA and microbiota composition, may require days to weeks [95,99-103]. This makes the gut axis one of the slower recovery-control axes and supports longer recovery timing because barrier repair, immune reprogramming, sIgA containment, and microbiota restructuring do not usually behave like rapid cytokine suppression.

Tumor Necrosis Factor-Like Cytokine 1A (TL1A) Pathway

TL1A is included here as an emerging immune-amplification axis that may become important in selected LGCI phenotypes. TL1A signals through death receptor 3 (DR3) and can support Th1 and Th17 responses, mucosal inflammation, gut-barrier injury, and tissue-inflammatory pathways, linking it to the wider NF-κB, IL-23/IL-17, and gut-axis loop network [104].

For RP, the relevant point is the timing of downstream recovery-control. TL1A-related inflammatory output may be limited by Th17-Treg-FoxP3 rebalancing, cholinergic reduction of upstream TNF-α and IL-1β input, gut-barrier recovery, and resolution signaling. These controls can require multihour to multiday windows, as described for the IL-23/IL-17 and gut-axis systems [91-93,95].

General Inflammatory Recovery Timing: Biological Background

Reported pathway timing was interpreted as directional support for extended biological response and recovery, not as evidence for a universal or clinical RP repetition interval. Because most available studies involved healthy subjects or experimental models not selected for low-threshold or slow-recovery biology, conservative starting intervals and response-guided adaptation remain necessary.

Beyond the pathway-specific brakes and recovery-control processes described above, general human inflammatory-resolution models support a 72-hour resolution reference window [105-108]. In an intradermal ultraviolet (UV)-killed *E. coli *model, bacterial clearance, granulocyte clearance, and catabolism of pro-inflammatory cytokines occurred by 72 hours. In the cited acute, localized inflammatory models, 72 hours represents a resolving-phase observation point, associated with declining polymorphonuclear neutrophil (PMN) numbers, changes in monocyte/macrophage phenotype, and enhanced pro-resolving lipid mediator signals, including D-series resolvins [107,109,110]. These findings provide a model-specific mechanistic reference point but do not establish a general recovery window for chronic systemic inflammation, human LGCI, or RP repetition. Throughout this review, established pathway biology is distinguished from mechanistic inference and RP-specific hypotheses; evidence supporting individual biological components is not considered evidence of the therapeutic effectiveness of the integrated RP framework.

LGCI Persistence

Established LGCI can persist through several linked mechanisms and sustaining drivers, including self-sustaining inflammatory loops, continuing cofactor exposure, LGCI-related damage and memory, and incomplete resolution. These mechanisms are separated below for clarity, but in living biology, they may overlap, reinforce each other, and keep the system closer to inflammatory reactivation.

Self-Sustaining Inflammatory Loops

**Figure 6:**
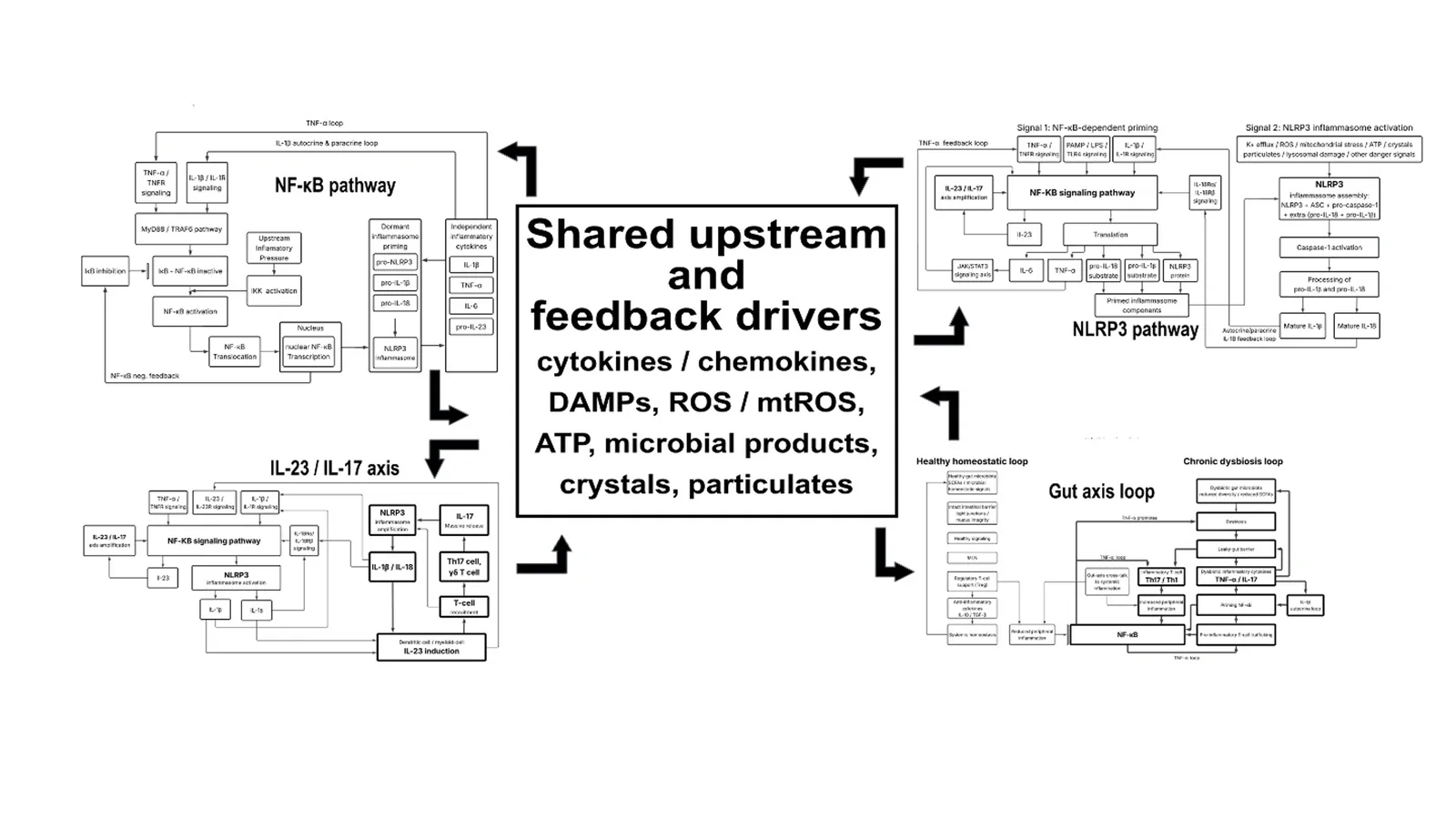
Overlapping self-reinforcing inflammatory loops in LGCI. Conceptual schematic of interacting NF-κB, NLRP3, IL-23/IL-17, and gut-axis inflammatory loops linked by shared upstream and feedback drivers: cytokines/chemokines, DAMPs, ROS/mtROS, ATP, microbial products, crystals, and particulates. An author-created conceptual schematic of established LGCI pathway-network biology. The cited sources support the major inflammatory axes represented in the schematic, including NF-κB activation by cytokine, microbial-product, PRR/TLR, and stress-related inputs; NF-κB-dependent cytokine-loop activity and NLRP3 priming; NLRP3 inflammasome activation by DAMPs, ion flux, ROS/mitochondrial stress, ATP, crystals, particulates, and lysosomal damage; IL-1β/IL-18 maturation and feedback; IL-23/IL-17 inflammatory amplification; dysbiosis, barrier dysfunction, microbial-product translocation, and gut-to-systemic inflammatory signaling; and allostatic or oxidative stress as reinforcing background pressure in chronic inflammatory states [1,3,10-13,16,54,65,94,95,98]. LGCI: low-grade chronic inflammation, DAMPs: damage-associated molecular patterns, ROS: reactive oxygen species, mtROS: mitochondrial reactive oxygen species, ATP: adenosine triphosphate, PRR: pattern-recognition receptor, TLR: Toll-like receptor.

LGCI is maintained by overlapping self-sustaining inflammatory loops (Figure 6). Cytokine feedback, NF-κB activity, inflammasome priming, oxidative stress, gut-barrier input, metabolic stress, and tissue signals can reinforce each other. This loop architecture helps explain why established LGCI can remain active even when an upstream trigger weakens, as illustrated in Figures 2-5 [54,68,69]. LGCI is therefore better viewed as a dense, interconnected inflammatory network.

Continuing Drivers, Cofactors

Continuing social, environmental, lifestyle, metabolic, microbial, or biological cofactors can contribute to LGCI over time and increase the probability of recurrent inflammatory activation. These cofactors are not necessarily the direct cause of established persistence by themselves; in many subjects, long-term exposure may be required before a persistent LGCI state develops. Once LGCI is established, cofactors can continue to add background pressure and can reactivate or worsen existing inflammatory loops [1]. Examples include visceral adiposity, ultra-processed dietary patterns, chronic mental or physical stress, physical inactivity, disrupted sleep, gut imbalance or barrier dysfunction, environmental toxins, tobacco smoke, excessive alcohol exposure, unresolved acute inflammation, chronic infection, or other phenotype-specific exposures [3].

LGCI-Created Damage and Memory

Long-standing LGCI can create damage, inflammatory memory, and tissue persistence that become part of the persistence problem. Oxidative and nitrative stress associated with inflammation can damage DNA, proteins, lipids, mitochondria, and membranes. Mitochondrial dysfunction, insufficient mitophagy, impaired efferocytosis, trained-immunity-like epigenetic memory, senescence-associated secretory signaling, barrier injury, endothelial dysfunction, extracellular-matrix remodeling, and fibrosis can keep tissues closer to inflammatory reactivation [111-114]. In cancer, DNA-damage-response pathways have become therapeutic targets, including through the development of DNA-damage-response inhibitors [115]. This cancer-specific evidence does not establish that such inhibitors treat damage attributed to LGCI or validate RP.

Incomplete Resolution and Reset

LGCI also persists because resolution and reset biology may be incomplete. Inflammation does not end only by reducing the trigger; it also requires active braking, cleanup, repair, and return to homeostasis. If these systems are weak, delayed, or repeatedly interrupted, the system can remain primed and low-threshold [60,111,116].

Current LGCI approaches and the unresolved timing variable

Anti-Inflammatory Medicine

These therapies can be highly effective in selected diseases and dominant-axis phenotypes. Their limitation for heterogeneous LGCI is not a lack of value, but that they usually act by ongoing suppression, blockade, or single-pathway modulation. When treatment stops, the suppressed axis can reactivate because the wider LGCI network, including overlapping inflammatory loops, may remain active. Therefore, these therapies are valuable, but they usually act as ongoing inflammatory control rather than durable multi-axis correction of heterogeneous LGCI; they usually do not remove the wider sustaining LGCI network [117-120].

Colchicine: Low-dose colchicine is valuable and often clinically successful in defined inflammatory phenotypes, including cardiovascular inflammatory risk, through effects on neutrophil activity and NLRP3-related inflammatory signaling. The LGCI limitation is that colchicine mainly targets a limited inflammatory axis, has a narrow therapeutic window, and is associated with risks [121].

Nonsteroidal anti-inflammatory drugs (NSAIDs): These drugs are valuable and often clinically successful in reducing pain and inflammatory symptoms by suppressing the cyclooxygenase/prostaglandin axis. Their LGCI limitation is that they mainly suppress one inflammatory axis and are associated with increased risks [122].

Glucocorticoids: These drugs suppress broad inflammatory transcription and also induce endogenous anti-inflammatory mediators such as IκB, dual-specificity phosphatase 1 (DUSP1), glucocorticoid-induced leucine zipper (GILZ), and annexin A1. Their advantage is strong and rapid anti-inflammatory activity. Their LGCI limitation is that they broadly suppress immune and repair biology, do not selectively restore durable multi-axis resolution, and are associated with risks [123].

Cytokine-targeted biologics: Anti-TNF, anti-IL-1β, anti-IL-17, anti-IL-23, and related antibody therapies reduce inflammatory output by neutralizing selected cytokines or receptors. Their advantage is strong efficacy when the dominant axis fits the disease phenotype. Their LGCI limitation is axis restriction and overlapping side effects [124].

Janus kinase (JAK) inhibitors: These inhibitors are valuable and often clinically successful because they block intracellular cytokine signaling downstream of multiple immune receptors. Their advantage is oral, multi-cytokine anti-inflammatory activity. Their LGCI limitation is that they suppress signaling broadly and carry safety concerns [125].

NLRP3 inhibitors: Emerging NLRP3 inhibitors aim to reduce inflammasome activation and IL-1β/IL-18 maturation. Their value lies in directly targeting a central inflammasome axis relevant to selected LGCI phenotypes. The pathological relevance of this target is illustrated by an LPS-induced inflamed-brain model in which NLRP3 was reported to exacerbate NETosis-associated neuroinflammation [126]; however, that study did not examine the safety or risks of NLRP3 inhibition. Their LGCI limitation is that NLRP3 inhibition does not by itself remove NF-κB priming, gut-barrier signals, crystals, mitochondrial stress, metabolic stress, tissue damage, or other upstream inputs.

Phosphodiesterase 4 (PDE4) inhibitors: PDE4 inhibitors increase intracellular cAMP and thereby strengthen cAMP-PKA-related anti-inflammatory signaling. Their clinical value is reinforcement of an endogenous intracellular brake. Their limitation is that their use is commonly limited by mechanism-based side effects [127].

Methotrexate/adenosine signaling: Low-dose methotrexate can exert anti-inflammatory effects partly through extracellular adenosine signaling and broader immune modulation. Its strength is the use of an endogenous anti-inflammatory signal. Its LGCI limitation is that it requires ongoing treatment and monitoring and is associated with some risks [128].

Fumarates/nuclear factor erythroid 2-related factor 2 (Nrf2) activation: The value of dimethyl fumarate and related fumarates is that they can activate Nrf2-linked antioxidant and cytoprotective defense biology and reduce inflammatory signaling. Their advantage is the support of endogenous cellular defense against oxidative-inflammatory stress. The LGCI limitation is that oxidative-stress control alone does not necessarily correct cytokine loops or complex LGCI biology. Risks have been reported [129].

Specialized pro-resolving mediator (SPM) approaches: Resolvins, lipoxins, protectins, maresins, and related pro-resolving mediators are especially relevant because they support active resolution rather than simple inflammatory blockade [130]. Their clinical advantage is conceptual alignment with cleanup, resolution, regulation of the immune system [20], and return toward homeostasis [19]. However, their LGCI limitation is that SPMs are short-lived, have low bioavailability, and may not localize well to inflamed tissue without specialized delivery systems [131]. This remains a major translational problem even if only small doses are needed [132].

SPM biology is not new. The first SPM-family mediator, lipoxin, was reported in 1984 [133]. The broader modern SPM field, including resolvins, protectins, and maresins, developed mainly from 2000 onward [134]. Today, the major translational problem remains unsolved [131], but research is ongoing.

Overall, the medicines discussed show that inflammatory axes and even endogenous brake systems can be pharmacologically controlled. Their shared limitation in heterogeneous LGCI is that they usually act through continuous suppression, blockade, or pathway modulation and often work on only a limited number of axes.

LGCI Trials

The difficulty of treating heterogeneous inflammatory biology is also illustrated by trials in which single-target anti-inflammatory strategies modulated biomarkers or pathway activity but failed to produce the expected clinical outcome.

In acute coronary syndrome, varespladib, an inhibitor of secretory phospholipase A2, had favorable effects on lipid and inflammatory markers but did not reduce recurrent cardiovascular events and was associated with increased myocardial infarction risk in VISTA-16 [135].

Darapladib, an inhibitor of lipoprotein-associated phospholipase A2, targeted a vascular inflammatory pathway but did not reduce major coronary events in SOLID-TIMI 52 [136,137]. Similarly, p38 mitogen-activated protein kinase (MAPK) inhibition with losmapimod reduced inflammatory markers in earlier studies but did not reduce major ischemic cardiovascular events after myocardial infarction in LATITUDE-TIMI 60 [138,139]. Together, these examples show that biomarker or pathway modulation is not the same as durable clinical benefit in complex inflammatory disease.

Merck reports tulisokibart, formerly PRA023, as an investigational humanized anti-TL1A monoclonal antibody, with Phase III ulcerative colitis (UC) induction endpoints met at 12 weeks, and the Phase II UC paper reported better clinical remission than placebo [140]. For Crohn’s disease, the available proof-of-concept study described it as potentially efficacious and well tolerated, with longer randomized trials needed [141].

Tulisokibart appears to be a promising inflammatory bowel disease (IBD) disease-control biologic for selected TL1A-associated phenotypes, especially where TL1A/DR3 signaling contributes to Th1/Th17 amplification, interferon gamma (IFN-γ), IL-17, mucosal inflammation, and fibrosis-related pathways. However, it remains a TL1A-suppressing, single-cytokine-targeting strategy. It may help manage selected IBD biology, but it is not a general LGCI intervention and does not by itself imply correction of the broader multidriver chronic inflammatory network.

Small-Dose Modalities

Small-dose pharmacological, nutritional, metabolic, biochemical, electromagnetic, mechanical, behavioral, or other low-burden inputs capable of producing measurable biological responses are relevant because biological responses are not always linear with input strength. Hormesis, microdosing, low-dose pharmacological or metabolic inputs, and sequential kinetic activation (SKA)-type low-dose signaling approaches belong to a broader small-drive design space, although evidence strength differs substantially by modality.

These approaches mainly address the amount or intensity of input [14,142-144]. They generally do not use long recovery intervals as part of the protocol. However, a time factor may still be present: when the dose is small, the stimulation may end before the next stimulation is given, although this interval is usually not treated as an active protocol variable.

Time-Dose Modalities

Several related fields show that biological response also depends on timing. Exercise physiology and rehabilitation medicine recognize that adaptation depends on stimulus intensity, repetition, fatigue, and recovery. Chronotherapy shows that treatment effects can depend on circadian phase and biological rhythms. Adaptive stress and preconditioning research also shows that the same stressor may have different effects depending on prior exposure and recovery state. Hormesis is relevant here in this physiological sense: many hormetic examples, such as exercise, dietary energy restriction, and ischemic preconditioning, use intermittent stress exposure followed by adaptive recovery [144-147]. Together, these disciplines and intervention frameworks show that timing can shape the response to repeated biological input [148].

Recovery-primed LGCI technology platform

RP Pathway Stimulation

**Figure 7:**
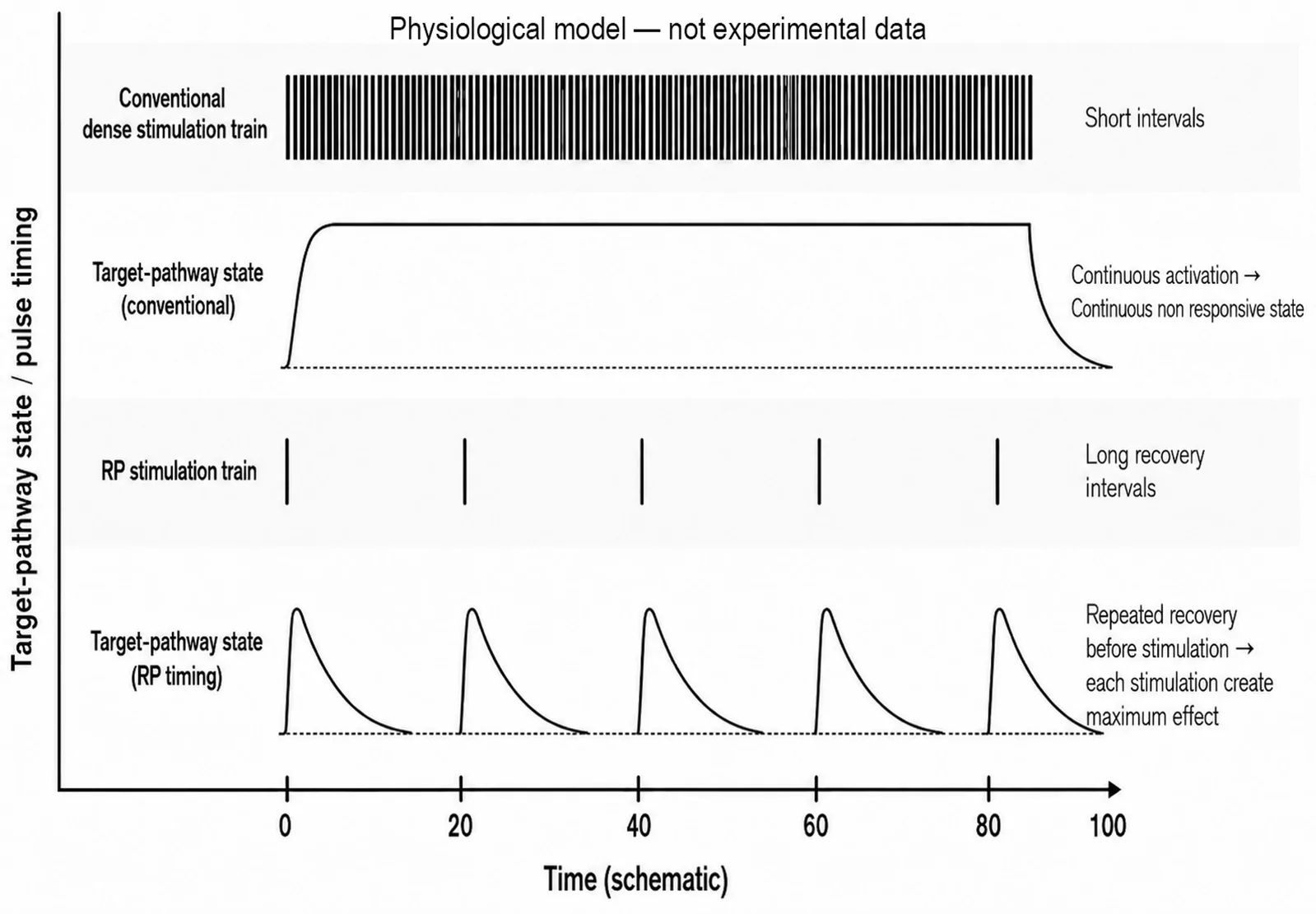
Conceptual model of target-pathway state under dense timing and RP timing. Author-created qualitative biological model illustrating that pathway activation may be followed by an altered, incompletely responsive, desensitized, or refractory-like state before recovery or reset restores useful responsiveness. The figure is not based on new experimental RP data, patient data, or numerical curve-fitting data; curves are schematic and not to scale. The biological response-state logic is based on published literature on inflammatory activation and resolution, non-resolving inflammation, allostatic load and recovery, temporal summation after repeated stimulation, and NF-κB feedback and oscillatory dynamics [35,59,60,116,149]. The dense-timing versus RP-timing comparison, including the placement of the next pulse after recovery-compatible responsiveness has returned, is author-proposed. RP: recovery primed stimulation, NF-κB: nuclear factor kappa B.

**Figure 8:**
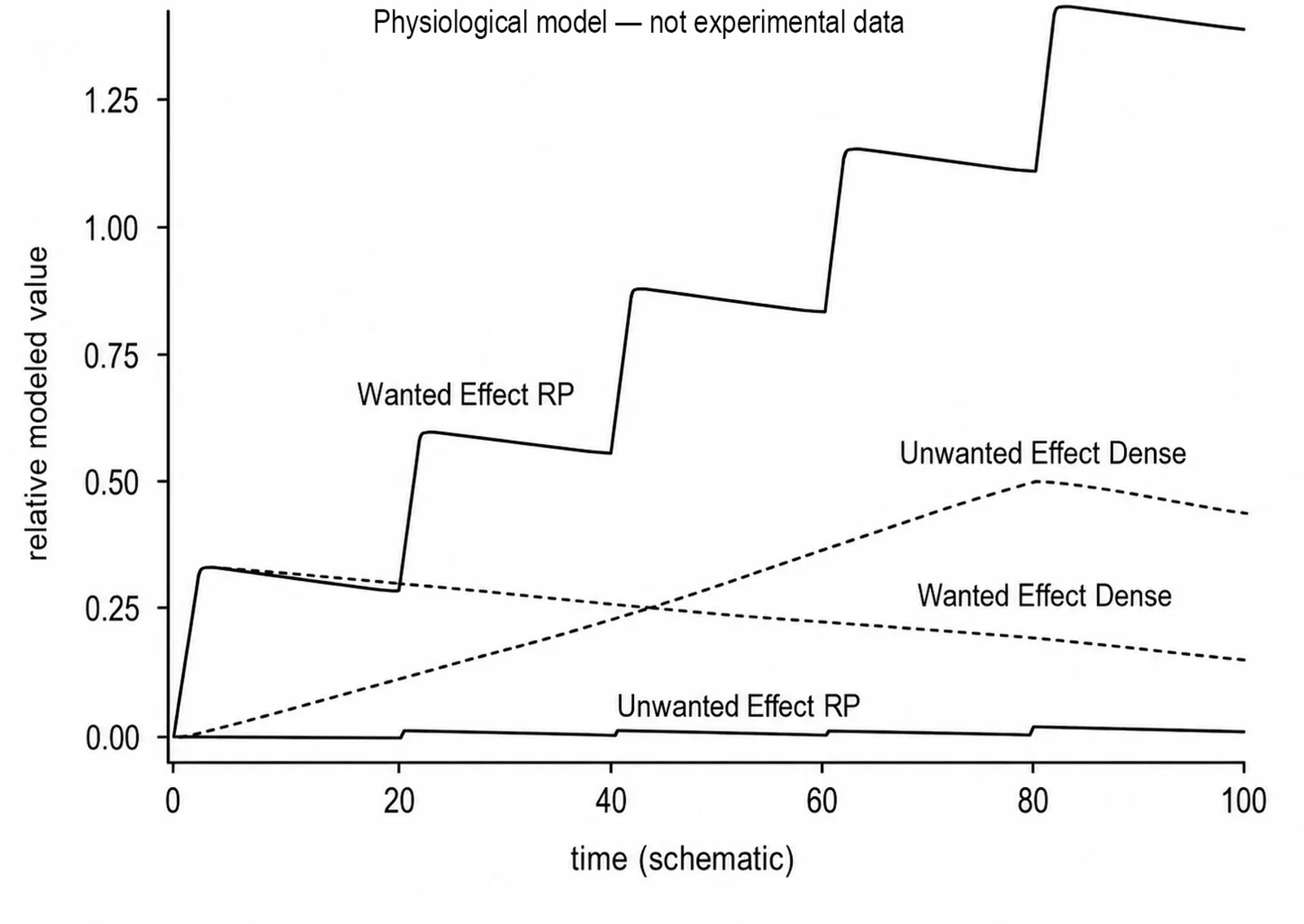
Conceptual model of wanted-effect and unwanted-effect behavior under dense timing and RP timing. Author-created qualitative biological model illustrating predicted wanted and unwanted effect behavior under the same pathway-state logic shown in Figure 7. The figure is not based on new experimental RP data, patient data, or numerical curve-fitting data; curves are schematic and not to scale. Dense timing is modeled as increasing the likelihood of incomplete recovery, temporal summation, adverse carryover, flare response, or unwanted cumulative burden. RP timing is modeled as reducing such carryover by allowing the active recovery phase to unfold before the next pulse. A central model prediction is separation of cumulative wanted effect: under dense timing, later pulses may arrive while the target pathway is incompletely responsive, so each later pulse may produce little or no renewed wanted effect; under RP timing, each pulse is modeled as arriving after recovery-compatible responsiveness has returned, allowing repeated renewed wanted effects over time. This timing logic is based on published literature on inflammatory resolution, non-resolving inflammation, allostatic load, temporal summation, and recovery-dependent biological responses [35,59,60,116,149]. The dense-timing versus RP-timing comparison and predicted RP-effect improvement under matched pulse drive are author-proposed. RP: recovery primed stimulation.

The figures in this section are physiological models, not experimental evidence (Figures 7, 8). They illustrate the RP pathway-state hypothesis: repeated stimulation may produce different biological effects depending on whether the next pulse is delivered before or after sufficient recovery of the relevant pathway system. In dense timing, closely spaced pulses are modeled as arriving before the target pathway has recovered, so later pulses may enter a persistently activated, altered, refractory, or incompletely responsive state. In RP timing, longer recovery intervals are modeled as allowing the target pathway to return toward a recovered, stimulation-responsive state before the next pulse.

Filling the Remaining LGCI Treatment-Design Gap

The preceding sections suggest that LGCI-relevant inflammatory braking, resolution, repair, and reset processes can require multiday windows. Current anti-inflammatory medicines can suppress selected inflammatory axes but often do not address the broader sustaining LGCI network. Small-dose approaches show that lower biological drive can be useful in some contexts, although the strength of evidence is inconsistent. Timing-dependent disciplines and intervention frameworks show that stimulation timing matters.

However, these approaches do not use the interval between repeated stimulations as an explicit protocol-control variable for improving wanted effects and limiting unwanted effects in LGCI. This is the remaining treatment-design gap addressed by RP.

Current anti-inflammatory medicines can suppress inflammatory output, but they usually do not coordinate recovery biology or restore the wider LGCI network toward durable resolution. RP is proposed to act on this functional network by timing repeated input to endogenous braking, resolution, repair, and reset windows. However, when self-sustaining inflammatory loops, established tissue damage, fibrosis, chronic infection, inflammatory memory, or continuing cofactor exposure keep reactivating LGCI, durable correction may require additional cofactor control, accelerators, or facilitators.

Inflammatory Braking as an RP-LGCI Tool

In this first practical proposal for moving LGCI toward homeostasis, NF-κB RP is used to illustrate the RP logic. In LGCI, persistent low-grade upstream pressure can maintain smoldering NF-κB activity while endogenous braking remains insufficient to restore stable homeostasis.

For the NF-κB example, a brief pulse with sufficient modality-specific pulse drive is proposed to raise canonical NF-κB activity above the smoldering LGCI baseline. Under the RP hypothesis, this may engage an endogenous negative-feedback response, including IκBα and IκBε feedback, without inducing sustained inflammatory activation [58,59,78,81].

The RP recovery interval then allows the induced brakes to be produced and to exert their temporary braking effect against the smoldering LGCI drive. These brakes are not expected to remain permanently elevated; they rise, act, and decay over time. The next pulse is timed by the chosen RP recovery interval, which is selected in relation to the effect window of the relevant long-acting brakes. The aim is to apply the next pulse when the delayed IκBε-related braking effect is approaching the end of its useful window, so that the following pulse can again induce a strong braking response relative to the smoldering LGCI state. At the same time, the long recovery interval is intended to avoid unwanted carryover between pulses [59].

**Figure 9:**
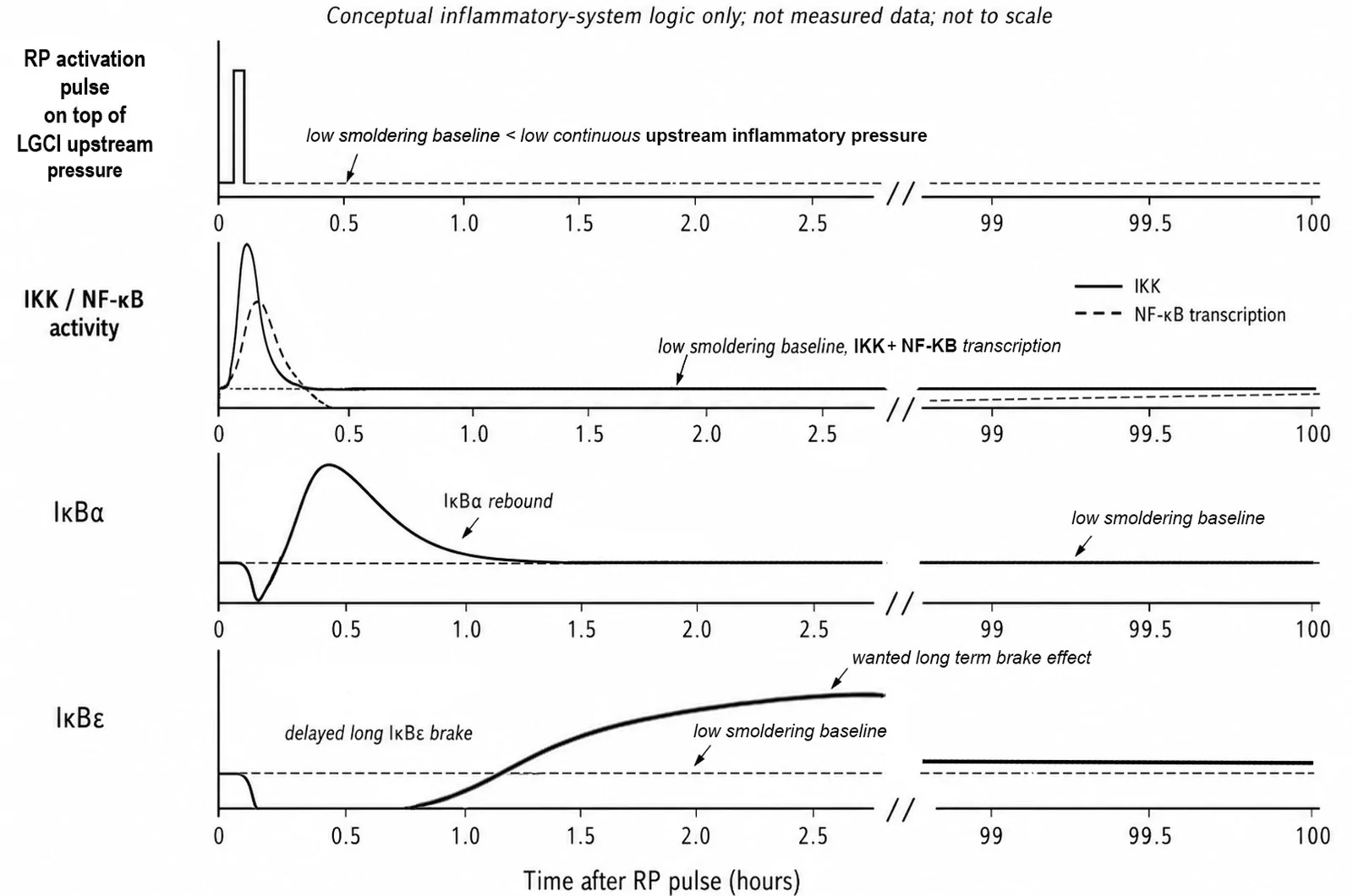
Conceptual model of RP-triggered NF-κB braking in smoldering LGCI. Author-created conceptual model of RP-triggered NF-κB braking in smoldering LGCI. The figure is a qualitative schematic, not plotted from new experimental RP data, patient data, or numerical curve-fitting data, and is not to scale. The qualitative sequence is based on published NF-κB/IκB feedback biology, including transient IKK/NF-κB activation, IκBα degradation/resynthesis and nuclear export, basal IκB/NF-κB homeostatic control, delayed IκBε negative feedback controlling NF-κB oscillations, signaling dynamics, and inflammatory gene expression, and prolonged post-activation IκBε detectability in an experimental immune-cell model [58,59,70,71,75,78,81]. The RP pulse, smoldering-LGCI baseline, and recovery-compatible repetition logic are author-proposed. RP: recovery primed stimulation.

Figure 9 illustrates the proposed RP logic in smoldering LGCI. A brief RP pulse transiently raises IKK and NF-κB transcription, after which early IκBα action and a delayed IκBε brake temporarily shift the NF-κB system away from the smoldering inflammatory baseline.

Inflammatory Resolution Through RP-Activated Recovery-Control Pathways

In this second practical proposal for using RP to move LGCI toward homeostasis, specialized pro-resolving mediator (SPM) biology is used to further illustrate the RP logic. SPM-related resolution biology can be engaged by both biochemical and biophysical inputs, and the SPM literature specifically describes active inflammation resolution rather than passive decay [19,150-153].

As discussed above, a 72-hour interval is a viable human inflammatory-resolution reference window. This makes optimized SPM-related RP activation compatible with the endogenous RP brake model and again highlights the importance of long RP recovery intervals.

SPM-related resolution biology may be engaged by a direct or indirect RP route. On the direct route, an RP input may engage lipid-mediator class switching and the lipoxin, resolvin, protectin, and maresin branches of the SPM network. In the indirect route, the RP stimulation, whether biochemical or biophysical, may create a controlled, temporary inflammatory response that supports lipid-mediator class switching and later SPM production.

The exogenous SPM translation problem is not limited to one delivery system. Recent reviews describe rapid degradation or metabolic inactivation, low bioavailability, poor localization to inflamed tissues, limited delivery strategies, and unresolved pharmacological parameters as continuing barriers to clinical translation of SPMs and SPM analogues [131,154].

**Figure 10:**
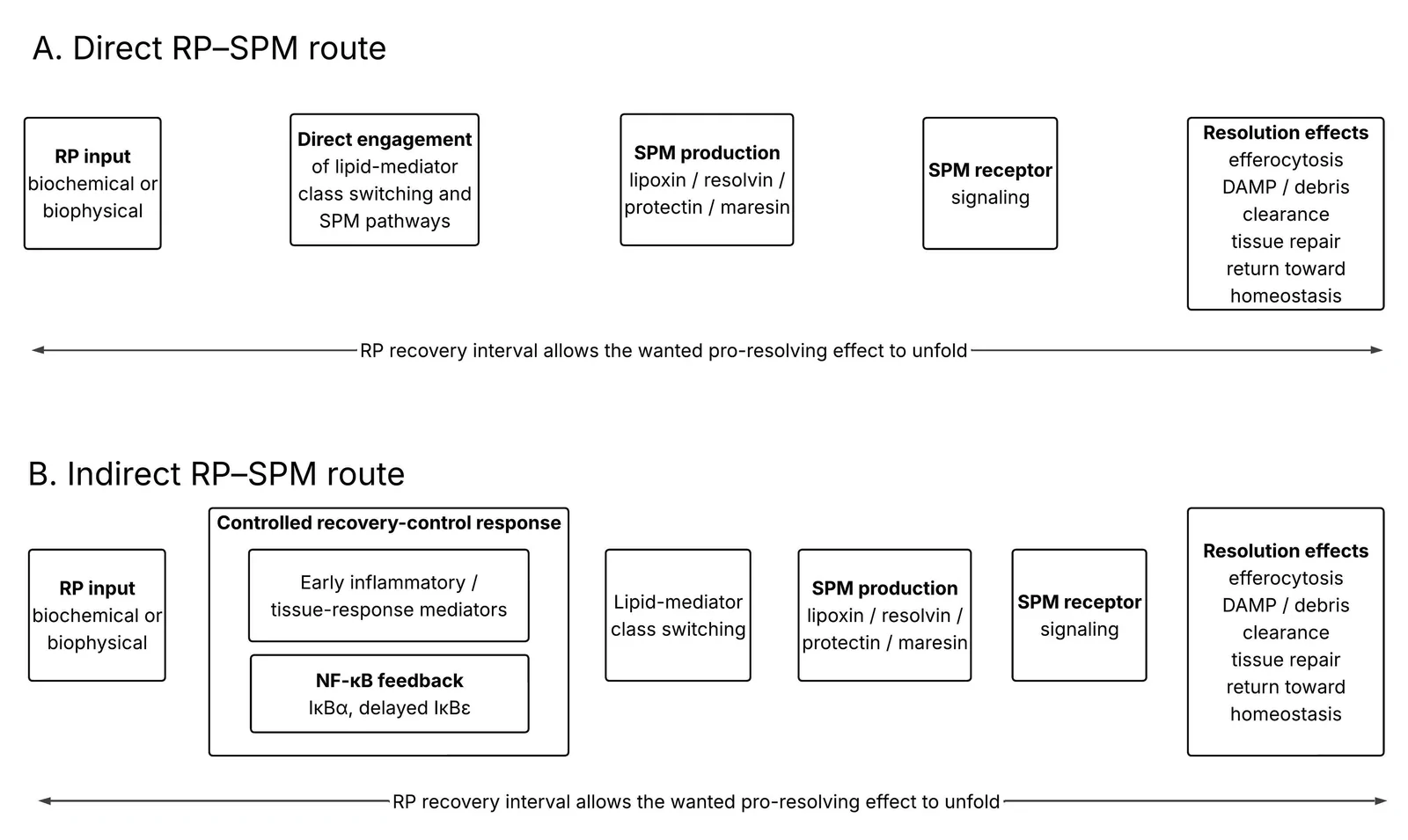
Conceptual model of direct and indirect RP engagement of SPM-related resolution biology. Author-created qualitative biological model illustrating the hypothesis that RP may engage specialized pro-resolving mediator (SPM)-related resolution biology through direct or indirect routes. In the direct route, an RP input may engage lipid-mediator class switching or related biochemical pathways that generate lipoxins, resolvins, protectins, maresins, or related pro-resolving mediators. This route is based on published evidence that endogenous lipid-mediator class switching and SPM-family mediator generation can occur during the inflammatory-resolution program and can be influenced by biochemical substrate and enzymatic context [132-134,150,153,155,156]. In the indirect route, an RP input may create a controlled temporary recovery-control response that is followed by endogenous resolution signaling, including SPM-related mediator production. This route is based on human inflammatory-resolution models showing resolving lipid-mediator signals during the resolution phase, and on intervention studies showing that physiological or neurostimulatory inputs can promote SPM-related resolution biology [107,109,110,151,152]. The figure is not based on new RP experimental data, patient data, or numerical curve-fitting data; it is schematic and not to scale. The direct/indirect RP-route structure, recovery-compatible repetition, and placement of the next stimulation after the pro-resolving response have had time to unfold are author-proposed. RP: recovery primed stimulation, SPM: specialized pro-resolving mediator.

RP proposes a different route. Instead of delivering SPMs as the therapeutic product, RP aims to stimulate endogenous SPM-related resolution biology through a biochemical, biophysical, or combined input. The RP recovery interval then allows the induced pro-resolving response to unfold before the next stimulation. In this way, RP may address part of the SPM translation problem by shifting from exogenous SPM delivery to endogenous SPM activation plus recovery-timed repetition (Figure 10). 

Under the RP model, RP-engaged SPM-resolution biology is expected to support anti-inflammatory and pro-resolving actions during the long recovery interval. These actions include limiting further inflammatory-cell recruitment, promoting macrophage efferocytosis, supporting debris and microbial clearance, and helping tissue return toward homeostasis [116,155,156].

RP-Induced Biological Amplification

RP does not amplify the physical pulse itself. It creates a larger useful biological effect from a brief pulse by using the body’s own recovery-control biology.

A short RP pulse can transiently raise the targeted pathway above the smoldering LGCI baseline, after which endogenous feedback systems can produce a much longer and stronger wanted effect than the pulse duration itself. Figure 9 illustrates this logic for canonical NF-κB braking. A brief RP pulse induces a short IKK/NF-κB transcriptional response, followed by early IκBα rebound and delayed IκBε induction. In this example, the important RP effect is not the short IKK pulse but the longer IκBε braking window that follows it. This illustrates an RP amplification principle: a short, controlled input can generate a longer recovery-control effect. The necessary condition for this amplification effect is the use of long, appropriate recovery intervals. In RP, a small input can produce a large biological effect when it is timed to the endogenous recovery biology it is intended to engage.

RP Is an Endogenous LGCI Model

RP is not continuous immune suppression, cytokine or receptor neutralization, antibody therapy, intracellular cytokine-signaling blockade, or single-axis control. The core difference between conventional anti-inflammatory medicine and the proposed RP platform lies in their therapeutic strategies.

RP does not primarily treat LGCI by blocking inflammatory output or by forcing resolution directly. RP is proposed as an endogenous recovery-control method that uses controlled triggering and recovery-compatible timing to support natural braking, resolution, repair, and return toward homeostasis.

RP for Chronic Viral and Parasite Persistence

In chronic viral or intracellular parasite persistence, RP is not proposed as nonspecific immune stimulation. The intended logic is recovery-first immune reactivation. By reducing LGCI and exhaustion pressure, RP may support recovery of T-cell and natural killer (NK)-cell function, including IFN-γ competence. In a suitable phenotype, a controlled RP trigger may then induce a short NF-κB-linked host-defense pulse that, when licensed by IFN-γ-signal transducer and activator of transcription 1 (STAT1)-interferon regulatory factor 1 (IRF1) signaling, supports nitric oxide synthase 2/inducible nitric oxide synthase (NOS2/iNOS)-dependent antimicrobial or antiviral activity. Because iNOS-derived nitric oxide (NO) is double-edged, this pulse must remain time-limited and must be coupled to parallel resolution-control arms, including IκBε/A20-mediated NF-κB braking and SPM-mediated resolution, before recovery-compatible repetition is considered.

RP-Effect Markers

What RP-effect markers can be used: Marker selection must be LGCI phenotype-dependent. Because LGCI is low-grade and heterogeneous, typical inflammatory markers such as IL-6, TNF-α, C-reactive protein (CRP), or related cytokines may remain within the reference range, show weak changes, or fail to capture the dominant pathway in a given phenotype.

More LGCI-specific marker sets have been proposed, including CD8+ T-cell subsets, monocytes, natural killer cells, B cells, and CD4+ T-cell subsets [3]. Leptin is also often proposed to characterize LGCI [157].

Candidate RP-effect domains include downstream clinical, functional, autonomic, inflammatory, symptom-timing, and mechanism-relevant markers selected for the phenotype. Examples include time to a predefined improvement milestone, symptom trajectory, gait or equilibrium measures, activity tolerance, sleep or recovery measures, airway or sinus symptom burden, heart rate (HR), blood pressure (BP), heart rate variability (HRV), barrier-related markers, metabolic markers, and epigenetic markers such as DNA methylation changes.

RP-effect validation: Each study should define the RP effect before testing, according to the LGCI phenotype, intended mechanism, modality, observation window, comparator condition, and safety endpoints. Validation requires showing that the predefined RP effect is reproducible, sensitive to the tested variable under matched-variable conditions, and associated with predefined safety, biomarker, clinical, functional, or patient-centered outcomes.

Oscillation-Avoiding

Inflammatory recovery intervals relevant to RP are expected to be far longer than the relatively short oscillation periods of the inflammatory pathway. NF-κB oscillations are reported on an approximately 100-minute time scale [158], whereas NF-κB braking through IκBε-related recovery control can support multiday timing [58,59]. This suggests that, in the RP timing context, IκBε-related braking is expected to damp rather than reinforce NF-κB oscillatory activity.

RP Accelerators and Facilitators for LGCI

RP accelerators and facilitators are optional, phenotype-matched support components. They are added only when they reduce a defined bottleneck in the selected phenotype. Their role is supportive within RP; they are not proposed as standalone curative interventions for LGCI.

An RP accelerator is intended to reduce a limiting inflammatory or biological background-noise axis that may interfere with the RP effect. Accelerator candidates may target the following axes: metabolic system, gut barrier, NF-κB, IL-23/IL-17 axis, oxidative stress, NLRP3 priming, and endogenous sugar and polyol pathways [1,3,95,159].

An RP facilitator may support recovery capacity, repair capacity, autonomic stability, metabolic flexibility, gut-barrier recovery, reduction of inflammatory memory, cofactor control, or reduction of persistent damage. Facilitators may therefore be especially relevant when the goal is durable LGCI correction rather than ongoing LGCI management [160,161].

Examples of facilitators include movement, autonomic, sleep/circadian, gut-barrier/microbiome, metabolic, thermal, biophysical, bioelectronic, pharmacologic-metabolic, neuroimmune, senescence/inflammatory-memory, and advanced medical load-reduction approaches [55,160-178]. Of special interest as facilitators are endogenous pro-resolving lipid mediators activated by RP stimulation.

Each accelerator or facilitator should be selected according to phenotype, cofactors, safety limits, intended RP role, wanted effects, and unwanted effects. Its value should be judged by whether it improves recovery-interval efficiency, tolerability, cumulative burden, or multi-axis suitability.

LGCI Management Versus Durable Correction

**Figure 11:**
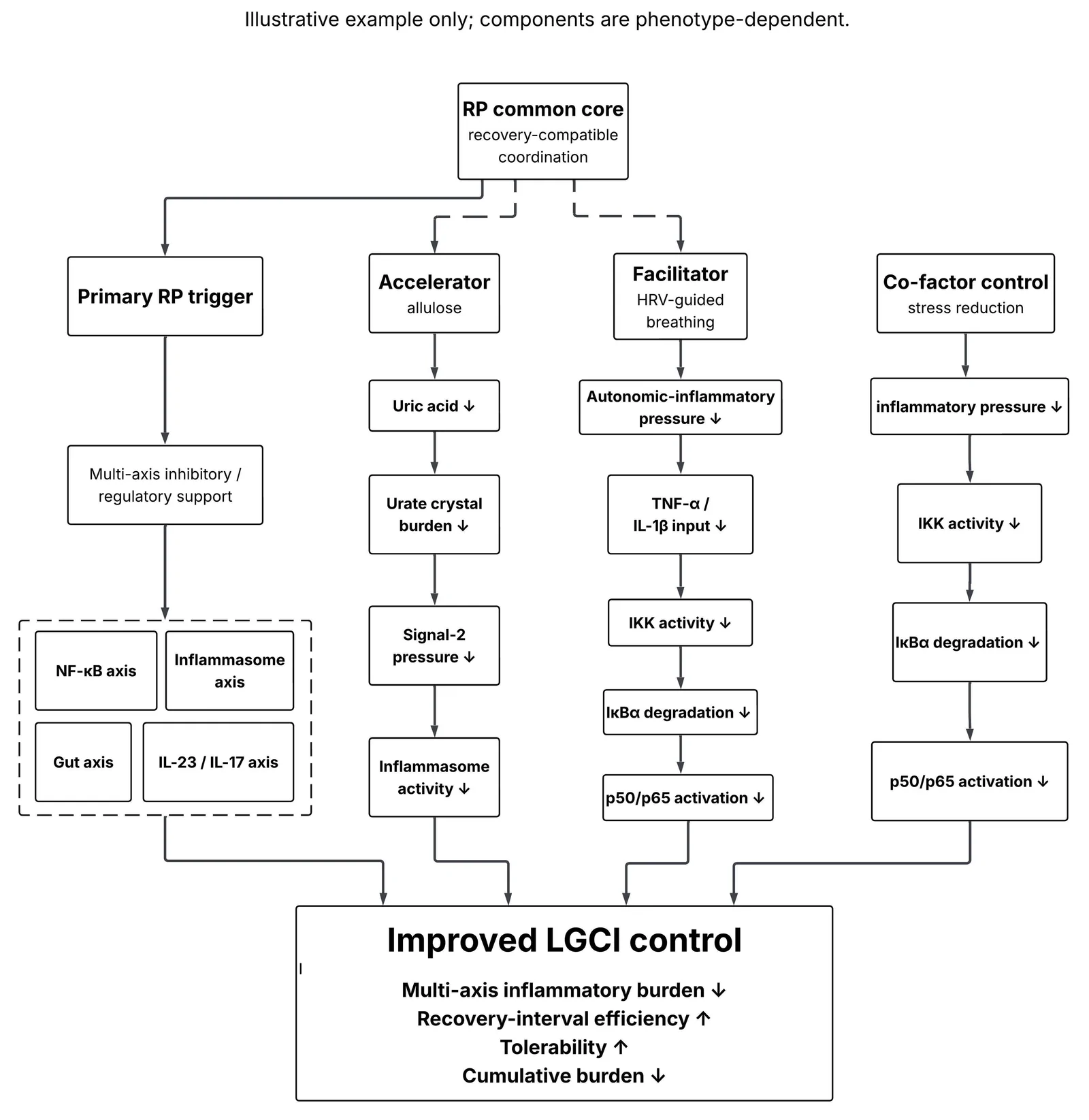
Example of coordinated recovery primed stimulation (RP) support in an illustrative inflammasome-dominant low-grade chronic inflammation (LGCI) phenotype. Author-created qualitative biological model illustrating how RP may be combined with phenotype-matched support components when one pathway axis or cofactor limits recovery. The example uses an inflammasome-dominant phenotype in which urate crystals or related danger signals may provide signal-2 activation of NLRP3 after NF-κB-dependent priming. In this architecture, the RP trigger is intended to engage a wanted recovery-control response; an accelerator is intended to reduce a limiting inflammatory or biological background-noise axis; a facilitator is intended to support recovery capacity, repair capacity, autonomic stability, metabolic flexibility, gut-barrier recovery, inflammatory-memory reduction, or resolution biology; and cofactor control is intended to reduce drivers that may reactivate LGCI loops. The figure is not based on new RP experimental data, patient data, or numerical curve-fitting data; the components are illustrative and do not define a clinical protocol. The NLRP3 inflammasome and danger-signal logic are based on published NLRP3 priming and activation biology, including NF-κB-dependent NLRP3 and pro-IL-1β priming and activation by danger signals such as crystals, ATP, mitochondrial stress, ROS, DAMPs, particulates, or lysosomal damage [11,13,54,68]. The cofactor and LGCI-network support logic is based on literature on chronic low-grade inflammation, metabolic inflammation, gut-barrier/systemic inflammation, allostatic load, exercise-related inflammatory modulation, circadian/sleep–immune regulation, and resolution biology [3,16,35,60,65,95,155]. The coordinated RP design, including the trigger–accelerator–facilitator–cofactor-control arrangement and its use for recovery-compatible repetition, is author-proposed. ATP: adenosine triphosphate, ROS: reactive oxygen species, DAMPs: damage-associated molecular patterns.

RP stimulation can be used for LGCI management or for durable LGCI correction. Because established LGCI can be persistent, durable correction may require long-term treatment and follow-up. During this period, RP may first function as LGCI management while progress toward durable correction is assessed. This process may be accelerated by adding phenotype-relevant accelerators or facilitators. Figure 11 illustrates this potential synergy using a selected inflammasome phenotype in which urate crystals provide signal-2 activation of NLRP3.

Inflammaging and Elderly LGCI Phenotypes

LGCI in older adults is not only “more inflammation” [2,3,179]. It is also shaped by accumulated molecular and tissue damage, mitochondrial dysfunction, cellular senescence, dysbiosis, immunosenescence, reduced repair capacity, and slower return to homeostasis [180-182]. This makes older adults a potentially important RP phenotype group because repeated stimulation is delivered into biology that may be more strongly primed, lower-threshold, and slower to recover. In this phenotype, the need for recovery-timed stimulation may therefore be greater.

The main proposed goal is not only lifespan extension but also improvement of health and quality of life. Relevant outcomes may include lower LGCI burden, better recovery, improved functional reserve, mobility, sleep, activity tolerance, autonomic stability, and reduced frailty-related decline [183-185].

RP Small-Drive for LGCI 

The LGCI small-drive RP applies recovery-timed stimulation to small-drive inputs. This is relevant in LGCI because primed, low-threshold biology may respond poorly to excessive pulse drive or dense repetition. Small-drive RP therefore aims to produce an RP effect with the lowest practical pulse drive and the lowest cumulative burden.

Because RP relies on biological amplification through endogenous recovery-control systems, a small input is not inherently a limitation, provided that it crosses the relevant biological activation threshold and the recovery interval allows the wanted effect to unfold. Small-drive RP is therefore not defined by the input being small. It is defined by the combination of a low pulse drive and a recovery-compatible interval. As a result, a small RP input drive does not make a protocol automatically safe, and it does not mean a small effect. Candidate small-drive inputs include low-dose, microdose, low-dilution, SKA-type, nutritional, metabolic, pharmacological, biochemical, biophysical, behavioral, or other low-burden inputs capable of producing a measurable biological response.

For clarity, this study does not rely on high-dilution preparations without plausible molecular content. Any small-dose input considered within RP must be capable of producing a measurable biological response.

Future research and protocol validation

RP Testable LGCI Predictions

**Figure 12:**
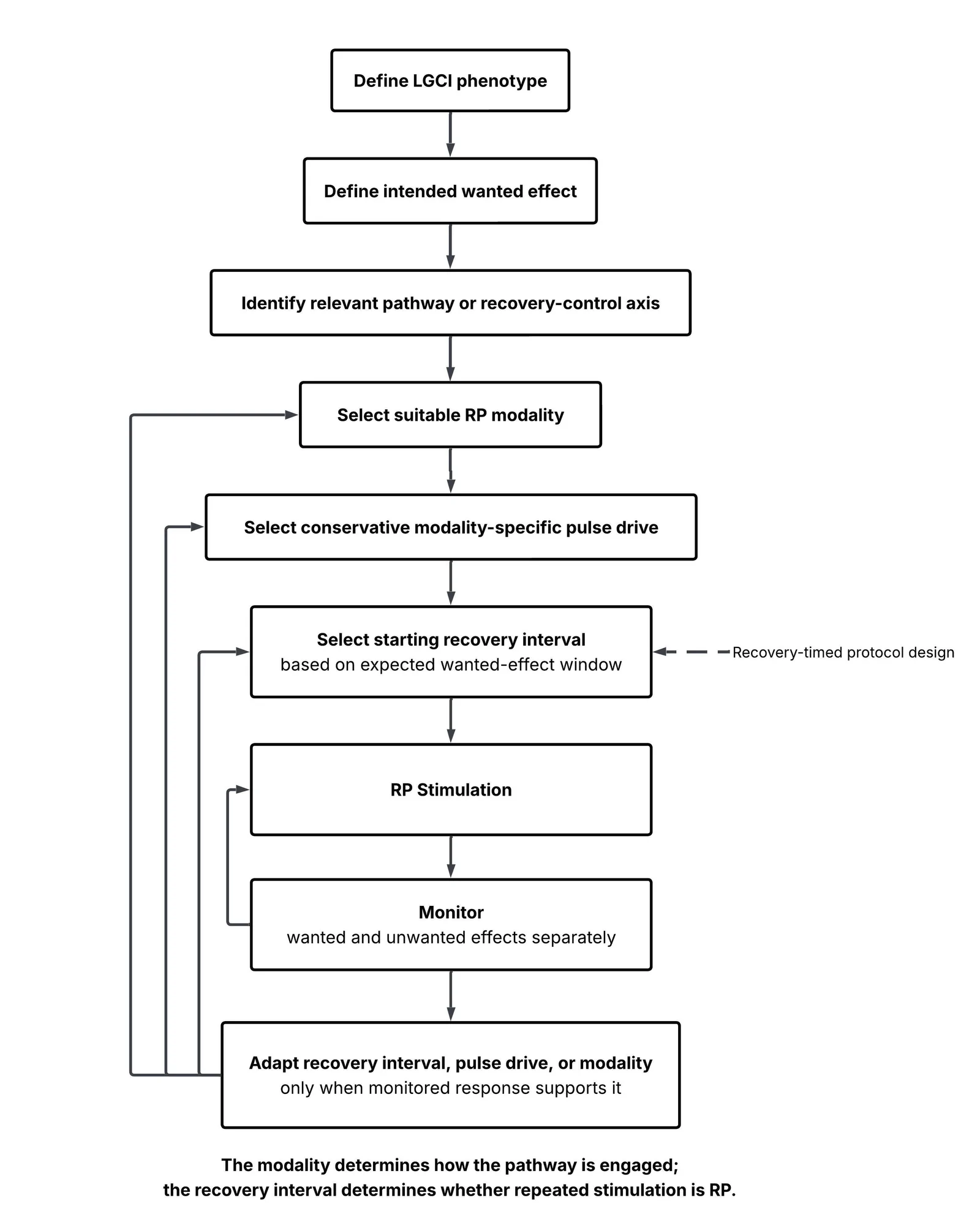
Practical RP design workflow. Author-created conceptual workflow illustrating how RP protocol design links LGCI phenotype, wanted effect, pathway target, modality selection, conservative pulse-drive selection, recovery-interval selection, monitoring, and adaptation into one recovery-compatible protocol-design sequence. The figure is not based on new experimental RP data, patient data, or numerical analysis. It summarizes the practical design logic proposed in this study: first define the phenotype and intended wanted effect; then select a relevant pathway target, a suitable modality, and a conservative starting pulse drive; then choose an initial recovery interval based on published pathway-timing and inflammatory-recovery biology; and finally adapt the protocol according to monitoring of wanted effects, unwanted effects, recovery status, and safety signals. The biological and clinical rationale for this workflow is based on published literature on LGCI heterogeneity, allostatic load, inflammatory resolution and non-resolution, recovery biology, and the need for phenotype-sensitive monitoring and adaptation [1,3,15,35,56,60,149,155]. The specific stepwise RP workflow and integration of these design elements into one protocol-development sequence are author-proposed. RP: recovery primed stimulation, LGCI: low-grade chronic inflammation.

The RP hypothesis generates testable predictions for comparing dense or non-recovery-timed repetition with a predefined RP-as-intended protocol. First, recovery-compatible repetition is predicted to increase predefined wanted effects compared with dense or non-RP repetition under matched and clearly reported input conditions. Second, RP timing is predicted to reduce the risk of stimulation-induced LGCI worsening compared with dense or non-recovery-timed repetition, as measured by predefined phenotype-relevant LGCI markers. Third, accelerators and facilitators are predicted to improve RP tolerance or durability only when they reduce limiting background noise, dominant inflammatory-axis activity, cofactor burden, or recovery bottlenecks in the selected phenotype (Figure 12).

Selecting and Monitoring RP Recovery Intervals in LGCI

The starting recovery interval should be conservative because recovery sufficiency cannot be predicted with certainty in LGCI, and the risk of starting too short is greater than the cost of starting too long. Published inflammatory recovery-window data have three limitations.

First, available physiological recovery data are incomplete for RP recovery-interval selection. Many pathways have been studied in depth, but downstream wanted-effect windows have received much less attention. In many studies, measurements stop while the effect is still strong, so the duration, decay profile, and useful end of the wanted recovery response remain unclear.

Second, inflammatory physiology in subjects with LGCI may recover more slowly than in the healthy subjects often used in physiological kinetics studies. Third, preliminary RP development observations suggest that shorter, approximately 72-hour-class intervals may be insufficient in LGCI-sensitive use and may need to be extended substantially.

The one-week and 10-12-day starting intervals proposed here are conservative protocol-development heuristics and author-proposed research hypotheses for average LGCI burden, not validated optimal RP recovery intervals, pooled estimates, or evidence-based clinical recommendations. They are based on available pathway-timing data, interpretation of those data, LGCI-specific considerations, and early RP development observations. For severe, reactive, or poor-recovery LGCI presentations, an initial interval of approximately 10-12 days is proposed as a conservative research starting hypothesis for prospective testing, not as an evidence-based clinical recommendation. The selected interval must be monitored and adapted when needed.

The timing values discussed in this review are not fixed RP recovery intervals. They identify only plausible wanted-effect windows and recovery-control processes that may inform protocol-development hypotheses. Actual RP interval selection remains dependent on phenotype, modality, pulse drive, predefined wanted effects, unwanted effects, and monitoring results.

Potential Recovery-Interval Problems and Risks

RP intervals can be nonoptimal for three main reasons: the recovery interval may be too short, too long, or also compatible with an unwanted pathway.

If the recovery interval is too short, the next pulse may arrive before recovery has sufficiently progressed. The system may still carry effects from the previous pulse, increasing the risk of temporal summation, adverse carryover, flare response, autonomic instability, or cumulative burden [35,60,186].

If the recovery interval is too long, the RP effect may be reduced because the wanted recovery response may have largely faded before the next pulse, and LGCI activity may begin to return before the next pulse is applied [60,107,149,155]. Delayed therapeutic responsiveness is unlikely to be an automatic consequence of long RP recovery intervals because these long intervals are active biological response periods, not empty rest periods [107,155].

Acute receptor desensitization, a rapid cellular mechanism that prevents overstimulation, is unlikely to be the main limitation for RP protocols using multiday recovery intervals [187-189]. Compensatory adaptation is also not expected as a direct consequence of correctly timed RP intervals. Compensatory biological burden is more consistent with frequent activation, failure to shut off stress-response systems, or persistent allostatic load, whereas RP separates pulses by recovery intervals intended to allow braking, resolution, and reset biology to unfold [15,35,155].

Rebound inflammatory activity is not expected simply because the RP interval is long. Rebound becomes a concern when the interval extends beyond the useful recovery window or when upstream LGCI drivers re-establish inflammatory pressure before the next pulse [60,116,149].

The main RP-specific safety risk is unwanted pathway amplification. If the selected recovery interval is also recovery-compatible for an unwanted pathway, repeated RP pulses may amplify unwanted effects even when the pulse drive is low. If wanted and unwanted effects remain linked, even after monitoring and adaptation, the protocol should be stopped unless a more specific RP modality or targeting strategy can produce the wanted effect without amplifying the unwanted effect.

Selecting specific phenotype recovery intervals: RP-specific protocol design may use published pathway data to organize candidate recovery intervals. Published pathway-timing data can help identify plausible starting intervals, but they do not validate RP, establish clinical protocols, or determine safety. Candidate intervals must therefore be treated as protocol-development hypotheses and adapted by phenotype, modality, pulse drive, wanted effects, unwanted effects, and monitoring results.

Importance of Monitoring and Adaptation

Optimizing RP effect: The optimal RP effect is not expected when the induced wanted effect has fully decayed. It is expected closer to a recovery-compatible timing point: late enough for the wanted effect to unfold, but not so late that the useful recovery effect has disappeared before the next stimulation.

Practical monitoring and adaptation of incomplete recovery: Adverse carryover may begin subtly before it becomes severe. Incomplete recovery may be suggested by autonomic, functional, inflammatory, sleep-related, or patient-reported changes after a pulse. Early warning signs may include dizziness, lightheadedness, disequilibrium, unusual anxiety, inner agitation, palpitations, tremor, headache, nausea, unusual fatigue, reduced activity tolerance, sleep disruption, elevated resting heart rate, unstable blood pressure, or HRV not returning toward the individual baseline.

When early warning signs appear, the stimulation protocol should be paused. Restart should occur only after the adverse response has resolved for a sufficient period and the protocol has been modified. Modification may include a longer recovery interval, lower pulse drive, shorter pulse width, or, when more than one pulse is used, fewer pulses or lower pulse density.

If adverse carryover recurs despite modification, the protocol should be further adjusted or stopped. If stimulation continues despite early warning signs, stronger signs of adverse carryover may include cold sweating, clammy skin, nausea, anxiety, marked orthostatic intolerance, presyncope, delayed malaise, symptom flare, pain flare, gut flare, airway or sinus flare, or worsening fatigue on the day after stimulation, indicating that protocol modification is needed.

If stimulation continues despite these stronger warning signs, stop signs may include vomiting, fainting, persistent dizziness, chest pain, shortness of breath, confusion, severe weakness, or marked instability of heart rate or blood pressure. These signs should not be treated as normal RP responses.

They indicate that the RP protocol, especially the recovery interval as a key RP protocol-control variable, is not appropriate for that subject at that time. These signs should trigger a protocol pause or discontinuation, appropriate medical evaluation, and protocol modification before treatment continuation.

Reassessment: If wanted effects are absent during monitoring, the protocol should not automatically be escalated. Pulse drive, phenotype suitability, target-pathway selection, and the RP recovery interval should first be reassessed before further stimulation is continued.

Early RP development context

During early RP development, pulsed electromagnetic field (PEMF) stimulation was used as a practical test-bench modality because its stimulation parameters and recovery intervals could be controlled and reported accurately. This was an exploratory implementation context for developing the RP hypothesis; it was not a controlled study, and no clinical efficacy conclusion can be drawn from it. It neither validates RP nor limits the proposed framework to electromagnetic stimulation.

Uncontrolled early development observations suggested that longer recovery intervals warranted formal investigation compared with denser repetition of the same input. These observations contributed to the proposed interval-selection logic, monitoring approach, safety boundaries, and testable predictions described in this study. They should be regarded as hypothesis-generating observations, not evidence of RP safety, efficacy, or an optimal repetition interval.

Safety and boundary conditions

Each RP modality requires its own exposure definition, contraindications, safety limits, monitoring plan, and validation before use. Safety cannot be generalized from one modality, pathway target, device, stimulation design, phenotype, or subject group to another.

In LGCI, the starting recovery interval should be chosen with a safety margin because recovery-interval sufficiency cannot be predicted with certainty. Pulse drive should also start conservatively and should be increased only when wanted effects, unwanted effects, and the recovery status support escalation.

Wanted and unwanted effects should be monitored separately. A wanted RP effect does not make a protocol acceptable if clinically meaningful unwanted effects are also produced. Monitoring, adaptation, and pausing rules should be predefined. If monitoring shows incomplete recovery, adverse carryover, flare response, autonomic instability, or cumulative burden, the protocol should be paused and adapted before restart.

Stopping when separation is not possible: If wanted effects and unwanted effects remain linked at the same recovery interval and cannot be separated by adapting the recovery interval, pulse drive, target pathway, or modality, the protocol should be stopped.

Limitations of the study

This study presents RP as a conceptual and testable framework. It does not report new experimental, clinical, biomarker, or patient-centered outcome data. No completed RP medical efficacy studies are reported here. Therefore, this study cannot establish RP efficacy, clinical benefit, disease modification, safety, optimal recovery intervals, or optimal protocols. These claims require pathway-, modality-, protocol-, and population-specific studies.

A major limitation is that many physiological studies describe pathway activation and early recovery kinetics but do not define the full downstream wanted-effect windows. Dedicated effect-window studies are therefore needed across different LGCI phenotypes and LGCI burden grades.

Another major limitation is LGCI heterogeneity. LGCI is treated here as an operational biological state, not as one disease entity. Different LGCI phenotypes may involve different dominant drivers, pathway loops, recovery kinetics, cofactors, tissue states, damage patterns, inflammatory memory, and safety limits. Therefore, RP timing cannot be generalized from one phenotype, modality, pathway target, device, stimulation design, or subject group to another.

A substantial part of the pathway-timing rationale is based on cellular studies, animal models, acute inflammation models, pathway-specific investigations, biological plausibility, and published pathway-timing data, not on validated RP dosing trials. These studies are useful for identifying candidate recovery-control processes and possible starting clocks, but they do not establish complete recovery intervals for complex human LGCI. Human LGCI is heterogeneous and may involve interacting pathway loops, cofactors, tissue damage, inflammatory memory, autonomic dysregulation, gut-axis involvement, medication effects, and phenotype-specific safety limits. Therefore, timing values derived from experimental or pathway-specific studies may under- or overestimate recovery timing in human LGCI. RP timing values provide candidate starting clocks, not definitive complete recovery intervals for patients with specific LGCI, and should therefore be treated as protocol hypotheses that require human validation.

Safety generalization is also a limitation. RP safety cannot be generalized from one modality, pathway target, device, stimulation design, phenotype, or subject group to another. Each RP modality has its own exposure definition, safety profile, contraindications, monitoring requirements, and validation needs. RP accelerators and facilitators also require separate evaluation because they may produce their own wanted effects, unwanted effects, interactions, or safety risks.

Finally, potential RP relevance to cancer-associated inflammatory biology remains speculative. Whether an RP effect occurs, is safe, or is useful in different cancer types remains to be investigated. The present article should therefore be read as a pathway-informed rationale for future testing, not as proof that RP is effective for LGCI or for any LGCI-associated disease field.

Scientific opportunity

RP creates a scientific opportunity to test recovery-compatible repetition as a protocol-control variable in low-threshold, slow-recovery LGCI biology. This research may be particularly relevant in phenotypes where LGCI contributes to persistence, poor recovery, symptom burden, or loss of physiological reserve across major chronic disease fields, including cardiometabolic disease, atherosclerotic disease, chronic kidney disease, fatty liver disease, neuroinflammatory and neurodegenerative conditions, musculoskeletal disease, gastrointestinal inflammation, cancer-associated inflammatory biology, and aging-related loss of resilience. The opportunity is to investigate whether recovery-timed intervention can reduce the LGCI component that contributes to persistence, poor recovery, symptom burden, or loss of physiological reserve in selected phenotypes.

## Conclusions

LGCI is a persistent, high-burden biological state for which no widely accepted general treatment strategy reliably resolves heterogeneous LGCI across phenotypes. The pathway review presented here supports the view that LGCI is difficult to address because inflammatory activity can be maintained by interacting loops, persistent inflammatory drivers, continuing cofactor exposure, low-threshold responsiveness, slow recovery, tissue damage, DNA damage, and inflammatory memory. The reviewed biology also suggests that LGCI-relevant wanted effects, including braking, resolution, repair, and reset processes, may require multiday windows to unfold. These observations provide a biological rationale for investigating recovery-compatible RP repetition after each pulse because dense or premature repetition may interrupt the wanted-effect window, increase unwanted carryover, or add inflammatory burden before recovery-control biology has completed its useful work.

RP is presented as a multimodal, multiaxis, multidisciplinary, hypothesis-generating intervention-development platform. Its primary design logic is controlled triggering of endogenous healing-related physiology followed by recovery-compatible repetition. The RP pulse is not the whole treatment; the biological response period after the pulse is central to the method because this is when induced braking, resolution, repair, and return toward homeostasis may unfold. RP therefore differs from conventional anti-inflammatory strategies that primarily rely on continuous inflammatory-output blockade, immune suppression, cytokine or receptor neutralization, intracellular cytokine-signaling blockade, or single-axis control. RP is intended to be developed as a physiology-guided framework that can be integrated with existing care pathways when appropriate, but its final role as adjunctive, supportive, or stand-alone requires indication-specific validation. The scale and persistence of LGCI, together with the lack of a widely accepted general treatment strategy, justify further pathway-specific, protocol-specific, and population-specific testing of the RP hypothesis. The proposed benefits of RP remain hypothetical and require confirmation through experimental and clinical validation studies.

## Appendices

Public survey-based examples of economic burden estimates for selected LGCI-related chronic disease fields are presented below.

**Table 1:** Public survey-based examples of economic-burden estimates for selected LGCI-related chronic disease fields. This table summarizes selected economic-burden estimates reported in publicly available surveys, reports, and published sources [36-52] for selected chronic disease fields in which LGCI-related mechanisms have been discussed in the literature; the economic values represent total disease-specific U.S. burden estimates and are not intended as LGCI-attributable cost estimates, but as an illustration of the scale of selected chronic condition fields in which inflammatory biology is implicated. The summary is provided only as contextual public-data survey material and is not a systematic review, primary survey, cost-effectiveness analysis, or pooled economic-burden estimate. Estimates are taken from publicly available surveys, reports, and published sources. The authors did not conduct the underlying surveys. LGCI: low-grade chronic inflammation, U.S.: United States.

Conditions with LGCI-related biology	Reported annual U.S. cost (US$ billions)	References
Heart disease and stroke	$419B	[40,43]
Diabetes	$413B	[40,41]
Alzheimer's disease	$360B	[40,45]
Arthritis, osteoarthritis and rheumatoid arthritis	$300B	[40,49]
Cancer	$240B	[40,44]
Nonalcoholic fatty liver disease	$292B	[48,52]
Obesity	$173B	[40,46]
Dental caries	$106B	[36,39,40,42]
Chronic kidney disease (Medicare costs only)	$95B	[40,47]
Parkinson’s disease (2017 estimate)	$52B	[50,51]
Inflammatory bowel disease	$50B	[37,38]
Approximate total for listed conditions	$2,500B	
